# A novel Cuprotosis-related signature predicts the prognosis and selects personal treatments for melanoma based on bioinformatics analysis

**DOI:** 10.3389/fonc.2023.1108128

**Published:** 2023-02-06

**Authors:** Bingqian Hu, Alphonse Houssou Hounye, Zheng Wang, Min Qi, Jianglin Zhang

**Affiliations:** ^1^Department of Dermatology, Xiangya Hospital, Central South University, Changsha, Hunan, China; ^2^School of Mathematics and Statistics, Central South University, Changsha, China; ^3^School of Computer Science, Hunan First Normal University, Changsha, China; ^4^Department of Plastic Surgery, Xiangya Hospital, Central South University, Changsha, China; ^5^Department of Dermatology, Shenzhen People’s Hospital (The Second Clinical Medical College, Jinan University; The First Affiliated Hospital, Southern University of Science and Technology), Shenzhen, Guangdong, China; ^6^Candidate Branch of National Clinical Research Center for Skin Diseases, Shenzhen People’s Hospital, Shenzhen, Guangdong, China

**Keywords:** bioinformatic, tumor immune microenvironment, melanoma, cuproptosis, immune–checkpoint inhibitors

## Abstract

**Background:**

Melanoma is a common and aggressive cutaneous malignancy characterized by poor prognosis and a high fatality rate. Recently, due to the application of Immune–checkpoint inhibitors (ICI) in melanoma treatment, melanoma patients’ prognosis has been tremendously improved. However, the treatment effect varies quite differently from patient to patient. In this study, we aim to construct and validate a Cuproptosis-related risk model to improve outcome prediction of ICIs in melanoma and divide patients into subtypes with different Cuproptosis-related genes.

**Methods:**

Here, according to differentially expressed genes from four melanoma datasets in GEO (Gene Expression Omnibus), and one in TCGA (The Cancer Genome Atlas) database, a novel signature was developed through LASSO and Cox regression analysis. We used 781 melanoma samples to examine the molecular subtypes associated with Cuproptosis-related genes and studied the related gene mutation and TME cell infiltration. Patients with melanoma can be divided into at least three subtypes based on gene expression profile. Survival pan-cancer analysis was also conducted for melanoma patients.

**Results:**

The Cuproptosis risk score can predict tumor immunity, subtype, survival, and drug sensitivity for melanoma. And Cuproptosis-associated subtypes can help predict therapeutic outcomes.

**Conclusion:**

Cuproptosis risk score is a promising potential biomarker in cancer diagnosis, molecular subtypes determination, TME cell infiltration characteristics, and therapy response prediction in melanoma patients.

## 1 Introduction

Melanoma, skin cancer of high malignancy, originates from melanocytes. The melanocytes are mainly located in the basal layer of the epidermis, which plays an essential role in the synthesis of melanin (1). The malignant transformation of melanocytes can cause melanoma and develops in the skin (2). Melanoma has a high metastatic potential, even a relatively small one. Regional lymph nodes and skin are the first and most common metastatic sites of melanoma, followed by distant visceral sites, like lungs, liver, bone, and so on (3). The incidence of melanoma is increasing year by year. New molecular subtypes for early melanoma diagnosis and prognosis have progressively become a field of interest (4). For example, Wu et al. divided 781 melanoma patients into different subtypes according to tumor-infiltrating immune cells (TIICs) and immune-related genes (IRGs). They found that the two groups prognoses are different (5). Whereas considerable progress has been made in molecular subtypes of melanoma, the prognosis prediction and therapeutic efficacy of melanoma patients remain unsatisfactory. In the studies that follow, it is of great interest to discover more molecular biological markers related to the prognosis of melanoma.

As an essential nutrient, copper plays a vital role in oxygen metabolism, oxygen radical detoxification and iron uptake (4). However, Copper can also cause impaired cellular functions and eventually cell death when there are excesses or deficiencies in the human body. An imbalance of copper homeostasis can cause irreversible damage to cells, even cell death. Studies showed that the imbalance in copper homeostasis can cause apoptosis and autophagy, through various mechanisms, including reactive oxygen species accumulation, proteasome inhibition, and anti-angiogenesis (6). Cuproptosis, a novel cell death manner, has been reported recently. A previous study found that Cuproptosis is critical in promoting the progression of a variety of tumors (7). Cuproptosis-related molecules are expected to be novel therapeutic targets for melanoma. Enormous Cuproptosis-related genes (CRGs) have been identified, some of which are positive regulation functions, and others are negative regulation functions. It is worthwhile to mention that the association between Cuproptosis and the prognosis of patients with melanoma remains unclear. Therefore, elucidating the detailed molecular characteristics of CRGs is conducive to predicting the prognosis of melanoma and precisive treatment.

In this study, on the basis of the expression of prognostic CRGs, 781 SKCM patients from four GEO melanoma cohorts and TCGA cohorts were divided into three Cuproptosis-related subtypes. We constructed the Cuprotosis score signatures of samples, building upon RNA transcripts, which could help predict prognosis in a number of patients with melanoma. Moreover, this study comprehensively analyzed various immune cell subsets using two computational algorithms: CIBERSORT and ESTIMATE. Furthermore, a prognostic nomogram that comprehensively combined Cuprotosis score signatures and the clinicopathological prognostic factors were constructed to predict individual prognosis. Our study demonstrated that Cuprotosis-related genes are attractive candidates as prognostic indicators.

## 2 Methods

### 2.1 Transcriptomic datasets, and survival information

**Figure S1** depicts a flowchart of the current work’s procedure. The Gene Expression Omnibus, The Cancer Genome Atlas (TCGA), and Genotype-Tissue Expression (GTEx) databases were used to provide gene expression (fragments per kilobase million, FPKM) and significant prognostic and clinicopathological data for SKCM. For the following analyses, four GEO melanoma cohorts (GSE19234, GSE65904, GSE78220, and GSE133713) and TCGA cohorts were collected. We retrieved the raw “CELL” files and adjusted the background and quantile standardization. The FPKM numbers of TCGA-Skin Cutaneous Melanoma (SKCM) were converted into transcripts per kilobase million (TPM) and were thought to be equivalent to those from microarrays (8).

The “Combat” technique was used to minimize batch effects after combining five datasets. We eliminated data from patients who did not have an OS; hence, 781 SKCM patients were included in the ensuing analyses. **Table S1** contains detailed information on these 781 SCKM patients. Age, gender, tumor site, TNM stage, follow-up period, and survival status were all clinical factors.

### 2.2 Consensus clustering analysis of CRGs

Ten CRGs were found in earlier papers (7). **Table S2** has complete information on these genes. To categorize patients into discrete molecular subgroups based on CRG expression, the R package “ConsensusClusterPlus” was used for consensus unsupervised clustering analysis. This clustering was done using the following criteria: First, the cumulative distribution function (CDF) curve progressively and gently grew. Second, there were no groups with small sample sizes. Finally, clustering boosted intra-group correlation while decreasing inter-group correlation. To study changes in CRGs in biological processes, gene set variation analysis (GSVA) was done using the MSigDB hallmark gene set (c2.cp.kegg.v7.2).

### 2.3 The relationship between subgroups and SKCM clinical characteristics and prognosis

We investigated the correlations between genetic subgroups, clinical and pathological features, and prognosis to assess the clinical utility of the subgroups found by consensus clustering. Age, gender, tumor site, and TNM stage were among the patient characteristics. Moreover, the variations in OS across subgroups were examined using Kaplan-Meier curves built by the R programs with “survival” and “survminer” packages.

Risk Score=


$$\sum_{i=1}^{n}\beta_{i}genei\left(expression\right)$$

where, *β_i_* is the parameter, and *gene_i_* is an expression of each gene.

### 2.4 TME, CLTA4, and PD-L1 molecular subgroup correlations in SKCM

Each patient’s immunological and stromal scores were evaluated using the ESTIMATE methodology. Furthermore, the CIBERSORT method was used to compute the percentages of 22 human immune cell types in each melanoma sample (9). A single-sample gene set enrichment analysis (ssGSEA) technique was also used to estimate the degree of immune cell infiltration in the SKCM TME (10). We also looked at the relationships between different subgroups and CLTA4 and PD-L1 expression.

### 2.5 Screening of DEGs and functional annotation

DEGs between Cuprotosis subgroups were found using the R package “EdgeR” with a fold-change of 2.0 and an adjusted p-value of<0.0001. Functional enrichment studies were performed on the DEGs using the “clusterprofiler” package in R to further investigate the probable functions of cuprotosis pattern-associated DEGs and uncover related gene functions and enriched pathways.

### 2.6 Establishment of the Cuprotosis-related prognostic CRG score

To measure the Cuprotosis patterns of the individual tumors, the Cuprotosis score was established. The DEGs were first applied to univariate Cox regression assessment to find those associated with SKCM OS. Second, using an unsupervised clustering approach based on the expression of prognostic CRGs, the patients were divided into three subgroup groups (Cuprotosis gene subgroup A, Cuprotosis gene subgroup B, and Cuprotosis gene subgroup C) for further study. Finally, all SCKM patients were randomly assigned to training (n = 452) and testing (n = 329) sets in a 70:30 ratio, and the former was utilized to calculate the Cuprotosis-related predictive CRG score. Although, using the R package “glmnet,” we built LASSO Cox regression models based on the expression status data of cuprotosis-related prognostic genes. The least absolute shrinkage and selection operator (LASSO) is a well-known approach for assessing survival data, and it is particularly effective for studying gene expression profiles with high dimensionality, small sample sizes, and highly significant variables (1,2). The “glmnet” package provided a series of models, with the value of the tuning parameter λ inversely related to the model’s complexity and deviation. The number of nonzero coefficients dropped as the value of the invisible λ rose from left to right. Ten-fold cross-validation was performed to discover the ideal λ values, and a value lambda = 0.0266 with log (λ) = -3.626844 was selected using minimal criterion. However, the value’s findings may vary significantly depending on the time of analysis. As a result, 10-fold cross-validation was performed up to 100 times, and cross-validated errors were averaged. Next, the cuprotosis-related prognostic genes derived from LASSO regression analysis were used in multivariate Cox regression analysis. The signatures were created by combining several cuprotosis-related prognostic genes, followed by the determination of the Akaike information criterion (AIC) value for each individual cuprotosis-related prognostic gene. Following that, the best prognostic signature was developed based on the lowest AIC value with the best goodness of fit.

A total of 452 patients in the training set were split into low-risk (CRG score< median value) and high-risk (CRG score *>* median value) groups and then conducted Kaplan- Meier survival analysis based on the median risk score. The “ggplot2” R software was then used to conduct principal component analysis (PCA). Furthermore, the testing and all sets were classified into low- and high-risk groups, with each subgroup conducted to Kaplan-Meier survival analysis and the creation of receiver operating characteristic (ROC) curves.

### 2.7 The prognostic CRG score’s clinical correlation and stratification analyses

Chi-square tests were employed to investigate the correlations between the CRG score and clinical variables (age, gender, tumor site, and TNM stage). We ran univariate and multivariate analyses on the training and testing sets to see whether risk scores were independent of other known clinicopathological variables. Furthermore, we conducted a stratified analysis to see whether the CRG score preserved its predictive potential in various subgroups based on age, gender, T stage, N stage, M stage, tumor stage, and tumor site.

### 2.8 Immune status and microsatellite instability were compared between high and low-risk groups

CIBERSORT was utilized to measure the quantity of 22 infiltrating immune cells in diverse samples from low- and high-risk cohorts to assess the ratios of TIICs in the TME. We investigated the relationships between the CRG score and the percentages of 22 invading immune cells. We also utilized boxplots to compare the amounts of immunological checkpoint expression between the low- and high-score cohorts. We also looked at the links between the two risk categories and MSI.

### 2.9 Drug sensitivity and mutation analysis

The mutation annotation format (MAF) from the TCGA database was developed using the “maftools” R package to differentiate the somatic mutations of SKCM patients into high- and low-risk categories. We also computed the tumor mutation burden (TMB) score for each SKCM subject in the two classes. We estimated the half maximal inhibitory concentration (IC50) values of chemotherapeutic medications routinely used to treat melanoma using the “pRRophetic” software to investigate variations in the therapeutic effects of chemotherapeutic agents in patients in the two classes.

### 2.10 Creating and validating a nomogram scoring scheme

Based on the results of the independent prognosis study, the clinical parameters and risk score were utilized to create a prediction nomogram using the “rms” package. Each variable in the nomogram scoring system was assigned a score, and the final score was calculated by summing the scores from all variables in each sample (11). The nomogram was evaluated using time-dependent ROC curves for 1-, 3-, and 5-year survivals. The nomogram calibration plots were utilized to show the predictive value between the anticipated 1-, 3-, and 5-year survival events and the practical actual results.

### 2.11 The suggested model’s pan-cancer analysis

We investigated the relationship between tumor mutational burden (TMB) and microsatellite instability (MSI) in 33 cancer species and determined whether they were upregulated or downregulated. Furthermore, in pan-cancer, a correlation study between risk score and TME as well as stemness indices was done.

### 2.12 Quantitative real-time PCR

DMEM medium supplemented containing 10% fetal bovine serum (FBS) was used to maintain A375 and HaCaT cell lines. These cells were cultured in a humidified chamber at 37°C with 5% CO_2_. The extraction of total RNA from cells was done by using a Trizol reagent (Sangon Biotech, China) according to the instructions supplied by the manufacturer. Ultraviolet absorption spectrometry was used to assess RNA quantity and quality. Evo M-MLV RT Mix Kit (Accurate Biotechnology, Hunan, China) from total RNA (1 μg) was used for cDNA synthesis, Evo M-MLV RT Mix Kit (Accurate Biotechnology, Hunan, China) from total RNA (1 μg) was used. Quantitative PCR (qPCR) was performed using the SYBR Green Premix Pro Taq HS qPCR Kit (AG11718, Accurate Biotechnology, Hunan, China). Primers shown in **Table S3** were used for amplifying products. The ΔΔcycle threshold method was used for quantifying the relative gene expression. GADPH acted as an endogenous control to normalize the expression level of relative gene expression. All results were obtained from at least three independent experiments with a minimum of three replicates per condition.

### 2.13 Statistical analysis

The K-M technique was used. To evaluate statistical significance, the log-rank test is utilized. R Computing Environment v4.1.2 was used for further analytical experiments. To study the prognosis of the estimate models for one, three, and five years, the ROC curve and AUC were exhibited using the “SurvivalROC” algorithm.

## 3 Results

### 3.1 Genetic and transcriptional alterations of CRGs in SKCM

**Figure 1:**
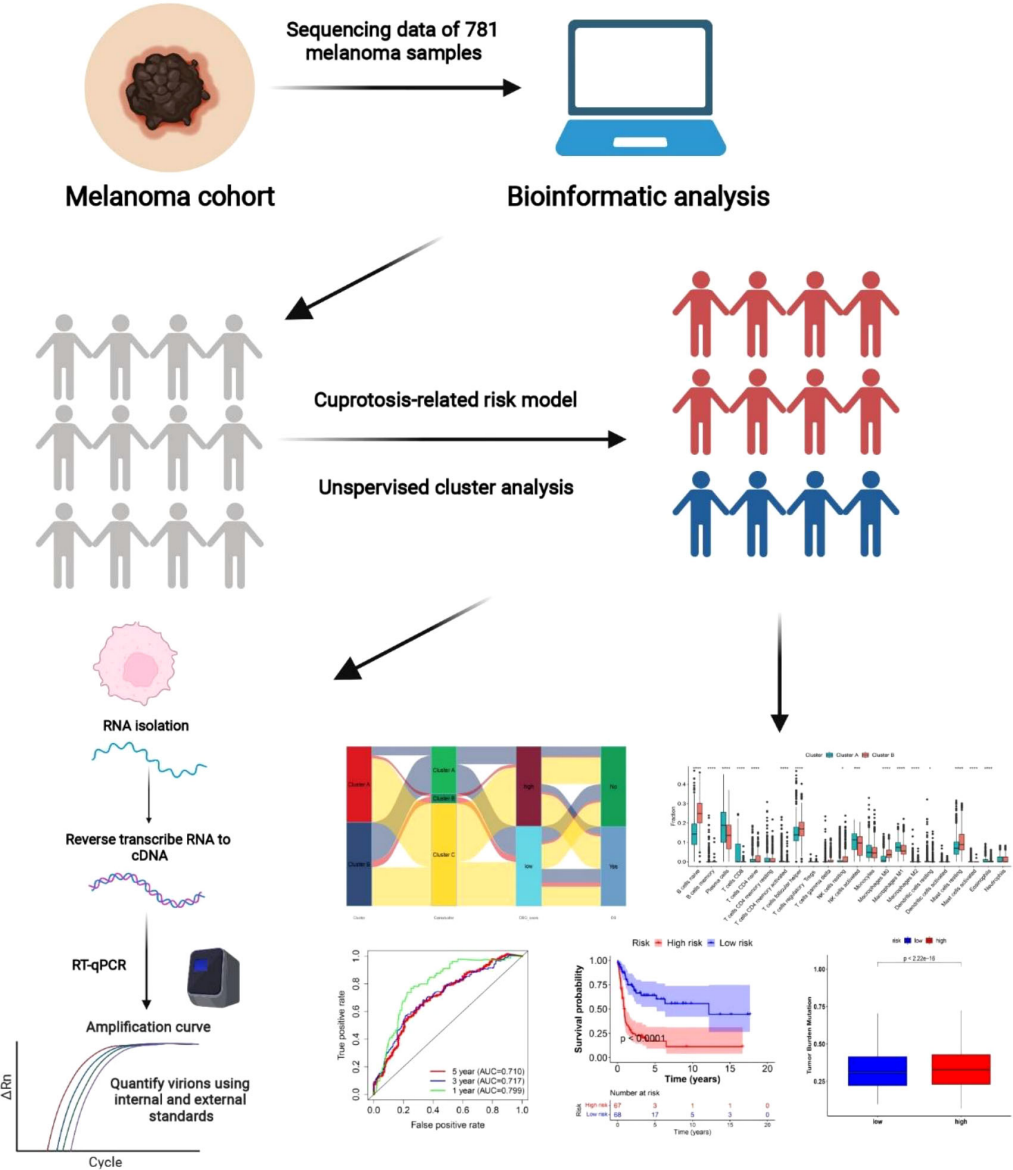
Graphical illustration of our present study.

**Figure 2:**
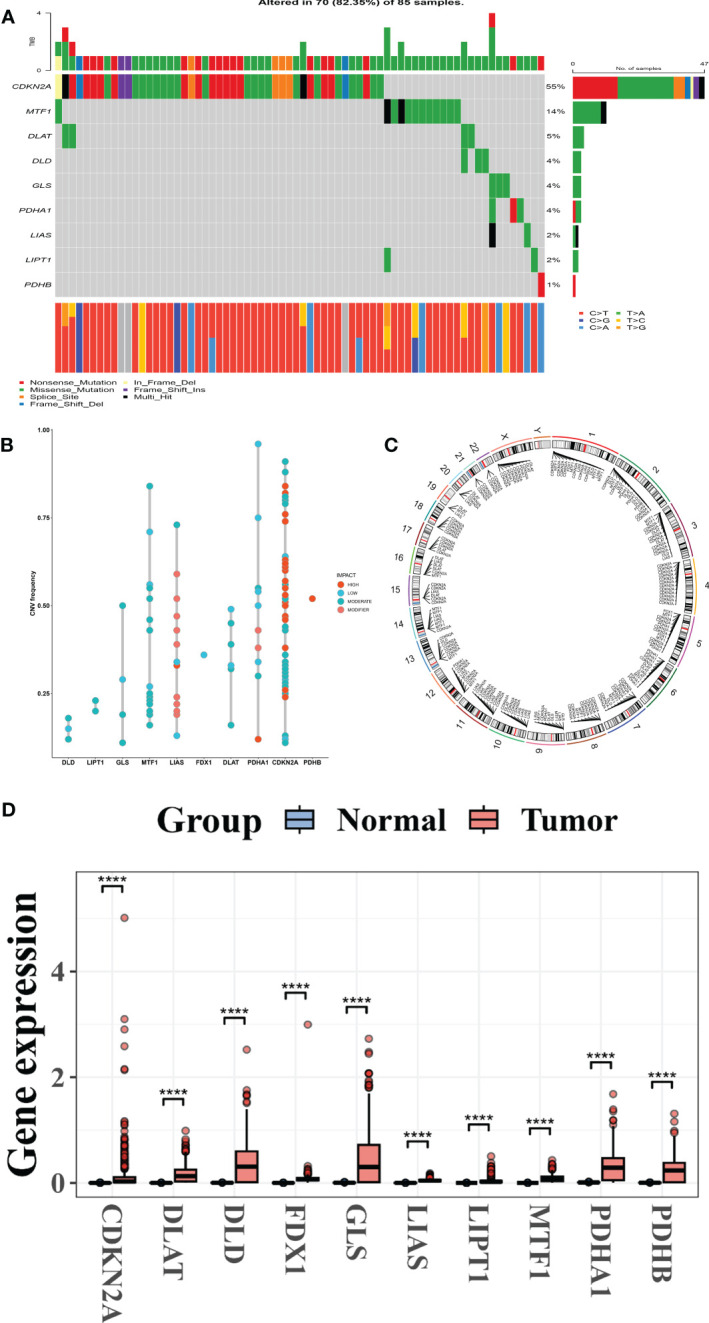
CRG genetic and transcription factors alterations in melanoma. **(A)** Mutation frequency range of ten CRGs in 85 SKCM cohorts from the TCGA patient. **(B)** Frequencies of CNV in high, low, moderate, and modifier CRGs. **(C)** CNV alterations in CRGs on 23 chromosomes. **(D)** Expression patterns of ten CRGs in normal and melanoma tissues. CRGs, Cuprotosis-related genes; SKCM, Skin Cutaneous Melanoma; TCGA, The Cancer Genome Atlas; CNV, copy number variant.

The **Figures 1** and **S1** depict the methodological approach used in this research. This research includes a total of ten CRGs. The SKCM cohort had a rather high mutation frequency, according to a summary study of the prevalence of somatic mutations in these 10 CRGs (**Figure 1A**). Of the 85 SKCM samples, 70 (85.35 percent) contained mutations in the CRGs (**Figure 2A**). CDKN2A exhibited the greatest mutation frequency (55%), followed by MTFF1, DLAT, DLD, GLS, PDHA1, LIAS, and others.

Following that, we investigated somatic copy number alterations in these CRGs and discovered common copy number alterations in all ten CRGs. GLS, MTF1, LIAS, FDX1, DLAT, PDHA1, CDKN2A, and PDHB all had widespread copy number variation (CNV) rises, whereas DLD and LIPT1 had CNV diminished (**Figure 2B**).

The locations of the CNV alterations in the CRGs on their respective chromosomes are depicted in (**Figure 2C**). We then evaluated by comparing the mRNA expression levels of SCKM and normal tissues and discovered that most CRG expression levels were significantly associated with CNV alteration. CRGs with low and moderate CNV, such as PDHA1, DLAT, GLS, and LIAS, were overexpressed in CRC comparison to those in normal melanoma samples, whereas CRGs with high CNV, such as PDHB, were significantly increased in melanoma samples (**Figure 2D**), implying that CNV may regulate CRG mRNA expression. While CNV can explain many observed changes in CRG expression, it is not the only factor involved in expression level regulation (12). Other factors that can influence gene expression include DNA methylation and transcription factors (13, 14). Our findings revealed a significant difference in the genetic landscape and expression levels of CRGs between melanoma and control samples, revealing a latent role for CRGs in melanoma tumorigenesis.

### 3.2 Identification of cuprotosis subgroups in SKCM

**Figure 3:**
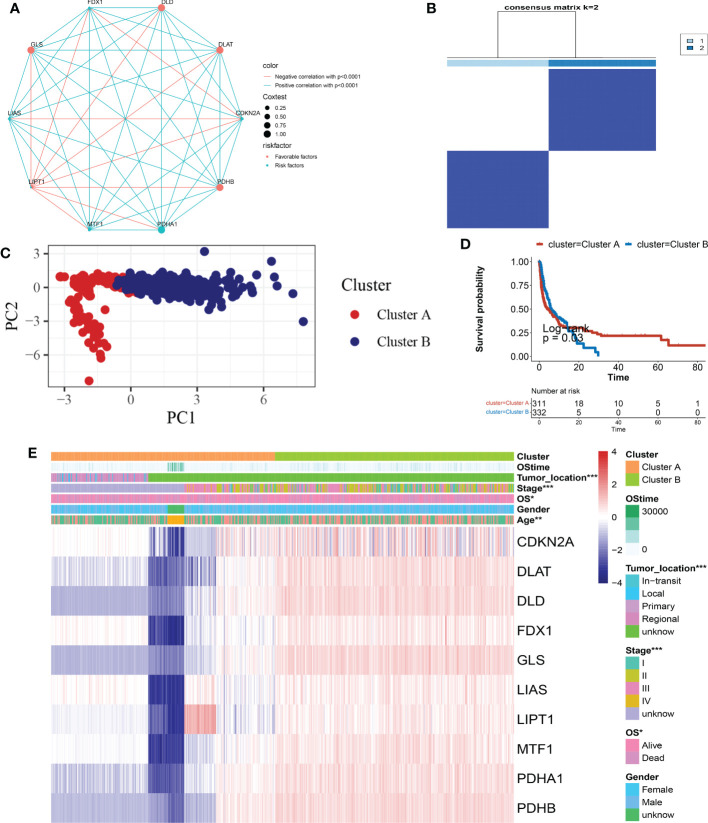
CRG subtypes, as well as clinical and pathological, and biological characteristics of 2 different subgroups of samples, were separated by consistent categorization. **(A)** Interactions between CRGs in SKCM. The interaction between CRGs is represented by the line connecting them, with the thickness of the line indicating the strength of the association between CRGs. Green represents negative correlations, while pink represents positive correlations. **(B)** Heatmap of the consensus matrix defining two clusters (k = 2) and their correlation area. **(C)** A PCA analysis reveals a significant difference in transcriptomes between the subgroups. **(D)** Univariate analysis of ten CRGs associated with OS time. **(E)** Clinicopathologic features and CRG expression levels differ between the two distinguishable subgroups. CRG, cuprotosis-related gene; SKCM, Skin Cutaneous Melanoma; PCA, principal components analysis; OS, overall survival.

Seven hundred and eighty-one patients from four eligible melanoma cohorts (TCGA-SKCM, GSE19234, GSE65904, GSE78220, and GSE133713) were included in our study for further analysis to fully understand the expression pattern of CRG involved in tumorigenesis. **Table S1** contains detailed information on the 781 melanoma patients. The prognostic values of ten CRGs in patients with SKCM were revealed by univariate Cox regression and Kaplan-Meier analysis (**Table S4**), and p< 0.05 was chosen as the filtering threshold. Following that, we ran a multivariate Cox regression analysis on five prognostic CRGs, three of which (FDX1, LIAS, and MTF1) were identified as independent potential predictors (**Table S5**). The comprehensive landscape of CRG interactions, regulator connections, and their prognostic value in melanoma patients was shown in a Cuprotosis network colored red (**Figure 3A** and **Table S6**). To investigate the expression properties of CRGs in melanoma, we used a consensus clustering technique on patients with melanoma based on the expression levels of the 10 CRGs (**Figure S2**). Our findings indicated that k = 2 showed up to be an efficient selection for sorting the entire cohort into subgroups A (n = 400) and B (n = 381) (**Figure 3B**). PCA analysis revealed significant differences in the transcription profiles of Cuprotosis between the subgroups (**Figure 3C**). The Kaplan-Meier curves revealed that patients with subtype A had a longer OS than patients with subtype B (log-rank test, p< 0.05; **Figure 3D**). Moreover, evaluations of the clinical and pathological features of various melanoma subtypes showed significant differences in CRG expression and clinical pathological characteristics (**Figure 3E**). As can be seen in **Figure 3E**, cluster A was associated with lower tumor location (p< 0.05), lower Stage (p< 0.05), and lower OS (p< 0.05) compared to cluster B.

### 3.3 TME characteristics in various subgroups

**Figure 4:**
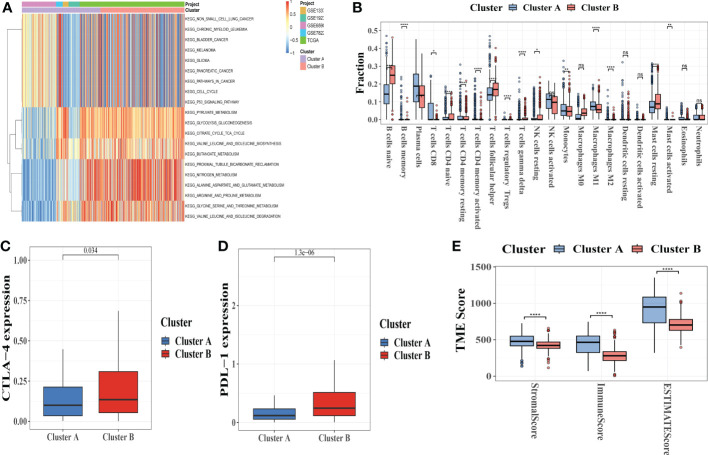
Correlations between tumor immune cell microenvironments and two types of melanoma. **(A)** GSVA of biological pathways between two distinct subtypes, with red representing activated pathways and blue representing inhibited pathways, respectively. **(B)** The presence of 22 different types of infiltrating immune cells in the two CRC subtypes. **(C, D)**CTLA-4 and PD-L1 expression levels in two melanoma subtypes. **(E)** Correlations between the two types of melanoma and the TME score. GSVA, gene set variation analysis; TME, tumor microenvironment. *p-value<0.05, **p-value<0.01, ***p-value<0.001, ****p<0.0001, ns is not significant.

Subtype A was significantly enriched in immunological fully-activated pathways, including cell cycle, p53 signaling pathway, pyruvate metabolism, and valine leucine and isoleucine degradation (**Figure 4A**; **Table S7**), according to GSVA enrichment analysis.

To examine the influence of CRGs in the TME of melanoma, we used the CIBERSORT algorithm (**Table S8**) to evaluate the correlations between the two subtypes and 22 human immune cell subsets of each melanoma specimen. We found significant differences in the infiltration of most immune cells between the two subgroups (**Figure 4B**).

Plasma cells, B cells memory, T cells CD8, CD4 memory-activated T cells, activated NK cells, monocytes, M1 and M2 macrophages, resting dendritic cells, and Eosinophils were clearly higher in subtype A than in subtype B, whereas B cells naive and T cells CD4 naive, T cells follicular helper, NK cells activated, M0 macrophages, mast cells activated had significantly lower infiltration in subtype A compared to subtype B. Similar fashion, analysis of two important immune checkpoints revealed that CLTA-4 and PD-L1 were more expressed in subtype B (**Figure 4C, D**).

We also used the ESTIMATE package to calculate the TME score (stromal score, immune score, and estimate score) for the two subtypes. Higher stromal or immune scores in the TME represented higher relative contents of stromal cells or immunocytes in the TME, whereas estimate scores indicated stromal or immune score aggregation in the TME. The results showed that patients with subgroup A had higher TME scores (**Figure 4E**).

### 3.4 DEG-based gene subtype identification

**Figure 5:**
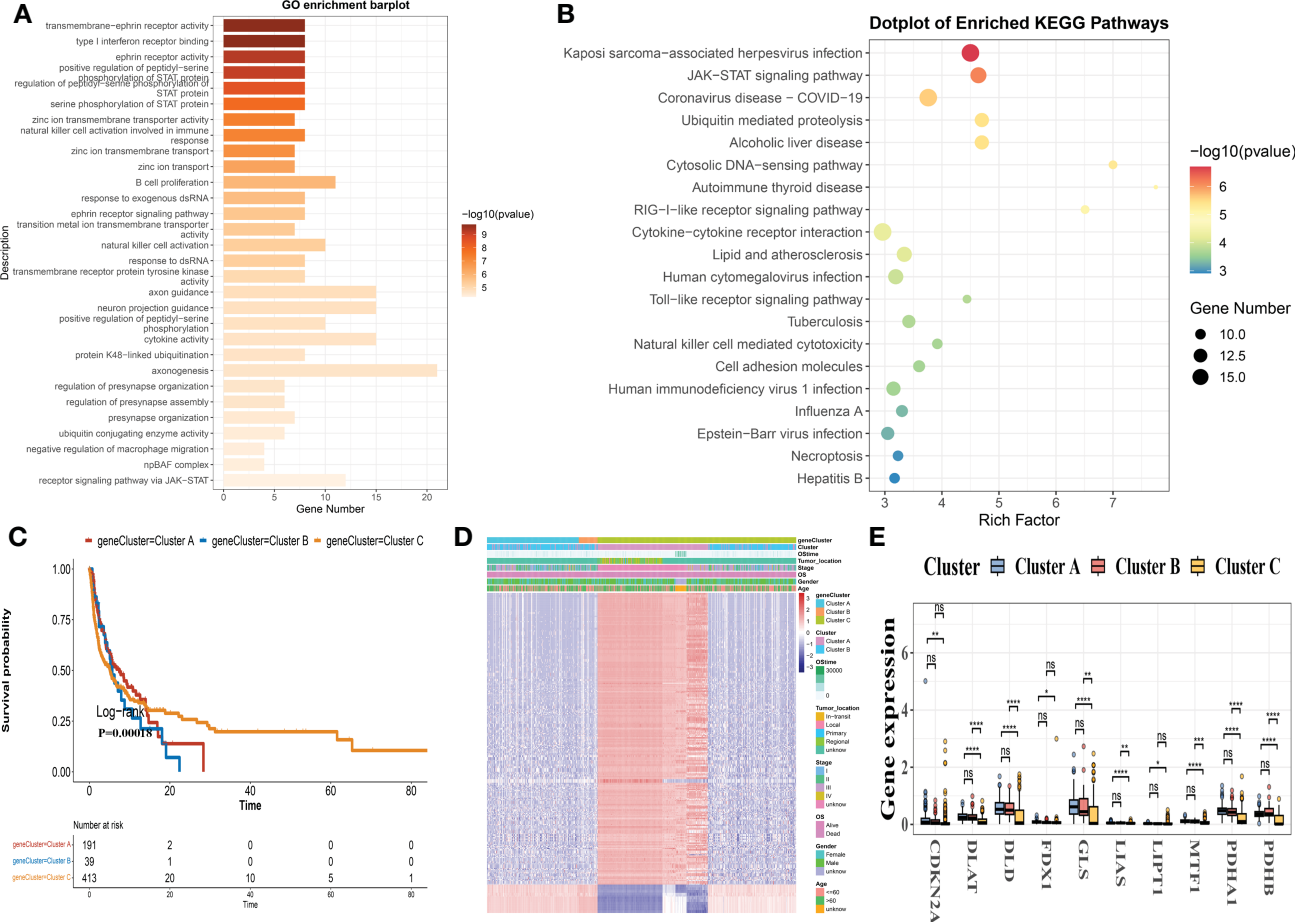
DEGs are used to identify gene subgroups. **(A, B)** GO and KEGG enrichment analyses of DEGs in two cuprotosis subgroups. **(C)** Kaplan-Meier curves for the two gene subgroups’ overall survival (log-rank tests, p< 0.001). **(D)** Associations between clinicopathologic characteristics and the two gene subgroups. **(E)** Expression differences in ten CRGs between the two gene subgroups. DEGs, differentially expressed genes; GO, Gene Ontology; KEGG, Kyoto Encyclopedia of Genes and Genomes; CRGs, Cuprotosis-related genes.

We distinguished 409 Cuprotosis subclass DEGs using the R package “EdgeR” and conducted functional enrichment analysis (**Figures 5A, B**; **Table S9**) to investigate the potential biological behavior of each Cuprotosis pattern. These Cuprotosis subcategory genes were significantly enriched in biological processes associated with immune function (**Figure 5A**). KEGG analysis revealed an enrichment of immune and cancer-related pathways (**Figure 5B**), implying that Cuprotosis is important in the immune regulation of the TME. We, therefore, used univariate Cox regression to determine the prognostic value of 350 subcategory genes and filtered out 227 genes associated with OS time (p< 0.05), which were used in the statistical evaluation (**Table S10**).

To validate this regulation mechanism further, a consensus-clustering algorithm was utilized to split patients into 3 genomic subclasses based on prognostic genes, namely, gene subclasses A-C (**Figure S3**). Kaplan-Meier curves revealed that patients with gene subtype B had the lowest OS, while patients with gene cluster C had a better OS (log-rank test, p< 0.001; **Figure 5C**). Furthermore, Cuprotosis gene subtype B patterns were linked to an advanced stage and a greater risk of OS (**Figure 5D**). The three Cuprotosis gene subgroups revealed substantial changes in CRG expression, which was consistent with the Cuprotosis patterns (**Figure 5E**).

### 3.5 Development and confirmation of the prognostic CRG score

**Figure 6:**
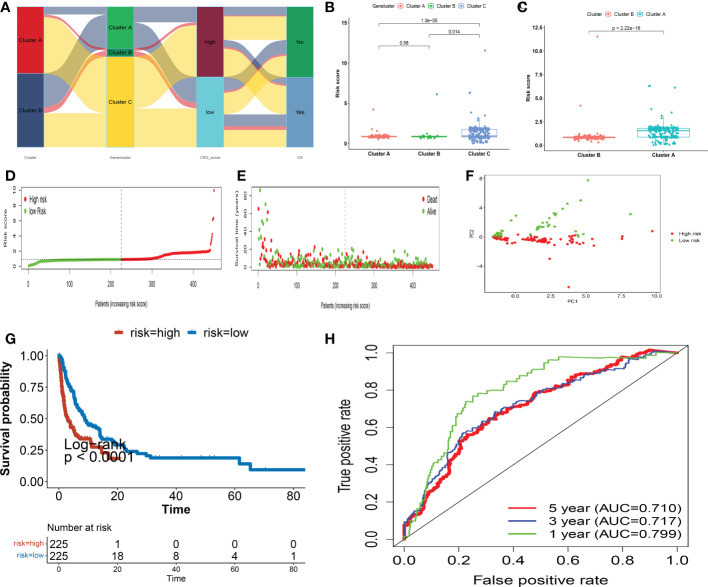
In the training set, the CRG score is constructed. **(A)** An alluvial diagram depicting subgroup distributions in groups with varying CRG scores and survival outcomes. **(B)** CRG score differences between gene subgroups. **(C)** CRG score differences between cuprotosis subgroups. **(D, E)** Dot and scatter plots display the distribution of CRG scores and patient survival status. **(F)** Prognostic signature-based PCA analysis. Patients at high and low risk are represented by red and steel blue dots, respectively. **(G)** Kaplan Meier analysis of the OS between the two groups. **(H)** ROC curves to predict the sensitivity and specificity of 1-, 3-, and 5-year survival according to the CRG score. PCA, principal component analysis; OS, overall survival; ROC, receiver operating characteristic.

The CRG score was calculated using the DEGs. **Figure 6A** depicts the subject distribution in the two Cuprotosis subgroups, three gene subgroups, and two CRG score classes. First, we utilized R “caret package” to randomly assign subjects to train (n = 452) and testing (n = 191) groups in a 7: 3 ratio.

To determine the best prognostic signature, LASSO and multivariate Cox analyses were conducted on 227 Cuprotosis subgroup-related prognostic DEGs. Following LASSO regression analysis, 15 OS-associated genes were retained based on the least partial likelihood of deviance (**Figures S4A, B**).

We afterward conducted multivariate Cox regression analysis on 15 OS-associated genes using the Akaike information criterion (AIC) value to obtain six (SUSD2, SCYL1, KLF9, GSPT1, KRT73, ZNF780A), which included four high-risk genes (SUSD2, KLF9, KRT73, and ZNF780A) and two low-risk genes (SCYL1, and GSPT1) (**Figure S4C**). The CRG score was calculated using the findings of the multivariate Cox regression analysis as follows:

Risk score= 0.0290 ∗ SUSD2 − 0.0276 ∗ SCY L1 + 0.0262 ∗ KLF 9 - 0.0263 ∗ GSPT 1 + 0.0266 ∗ KRT 73 + 0.0265 ∗ ZNF 780A

The CRG score differed significantly amongst Cuprotosis gene subtypes. Genecluster A had the lowest CRG score, whereas genecluster C had the highest score (**Figure 6B**). More crucially, subgroup A had a much higher CRG score than subgroup B. **Figure 6C** depicts the patterns of risk scores in the two subgroups. Subjects with a CRG score less than the median risk score were classified as low-risk (n = 225), while those with a CRG score larger than the median risk score were classified as high-risk (n = 225). The distribution plot of the risk of CRG score demonstrated that as CRG scores rose, survival times fell while OS rates increased (**Figure 6D, E**). PCA analysis revealed distinct dimensions between the low- and high- CRG score groups (**Figure 6F**). The Kaplan-Meier survival curves demonstrated that patients with low scores had considerably better overall survival than those with high scores (log-rank test, p< 0.001; **Figure 6G**). Furthermore, the 1-, 3-, and 5-year CRG score survival rates were indicated by AUC values of 0.799, 0.717, and 0.710, respectively (**Figure 6H**). We computed CRG scores across the internal (testing set) and one external validation group (GSE65904) (**Figures S5****-****6**) to verify the prognostic effect of the CRG score.

The subjects were also divided into low- and high-risk groups based on the training set formula. The CRG scores, patient survival status, and PCA demonstrating the variation tendencies of the low- and high-risk groups are shown in **Figures S5A, B**, and **S6A, B,** respectively. Survival analysis demonstrated that the low-risk group had a considerably better prognosis than the high-risk group (log-rank; p< 0.001; **Figures S5****-****6C**). The CRG score still had reasonably high AUC values (**Figures S5****-****6D**) when the 1-, 3-, and 5-year prognostic prediction classification efficiencies were examined, demonstrating that the CRG score had an outstanding capacity to predict the survival of melanoma patients.

### 3.6 The prognostic CRG score subjected to clinical correlation and stratification analysis

We investigated the link between the CRG score and several clinical features to determine the influence of the CRG score on clinical characteristics (age, sex, tumor location, and Stage). We found that patients in the stage IV subtype had substantially higher CRG scores than those in the stage I subgroup (p< 0.05; **Figure S7A**). To see whether this prognostic CRG score might predict OS independently in melanoma patients, we coupled clinical characteristics with the prognostic CRG score and ran univariate and multivariate analyses. The Stage and CRG scores in the training set indicated substantial differences, as shown in **Figures S7B, C**, with similar findings found in the testing (**Figures S7D, E**), and GSE65904(**Figures S7F, G**) groups.

Furthermore, a stratified analysis to determine whether the CRG score maintained its predictive ability in different subgroups, including age (= 60 and > 60 years), sex (female and male), tumor location (in-transit, local, primary, and regional), stage (stage I-II and stage III-IV), revealed that patients with high-risk scores had significantly lower OS compared to those with low-risk scores age (p< 0.01), sex (p = 0.0064 in women and p< 0.0001 in men), tumor location (p< 0.0001 in-transit, local, primary, and regional), stage (p< 0.001)(**Figure S8**).

### 3.7 TME and checkpoints compared amongst high-risk and low-risk classes

**Figure 7:**
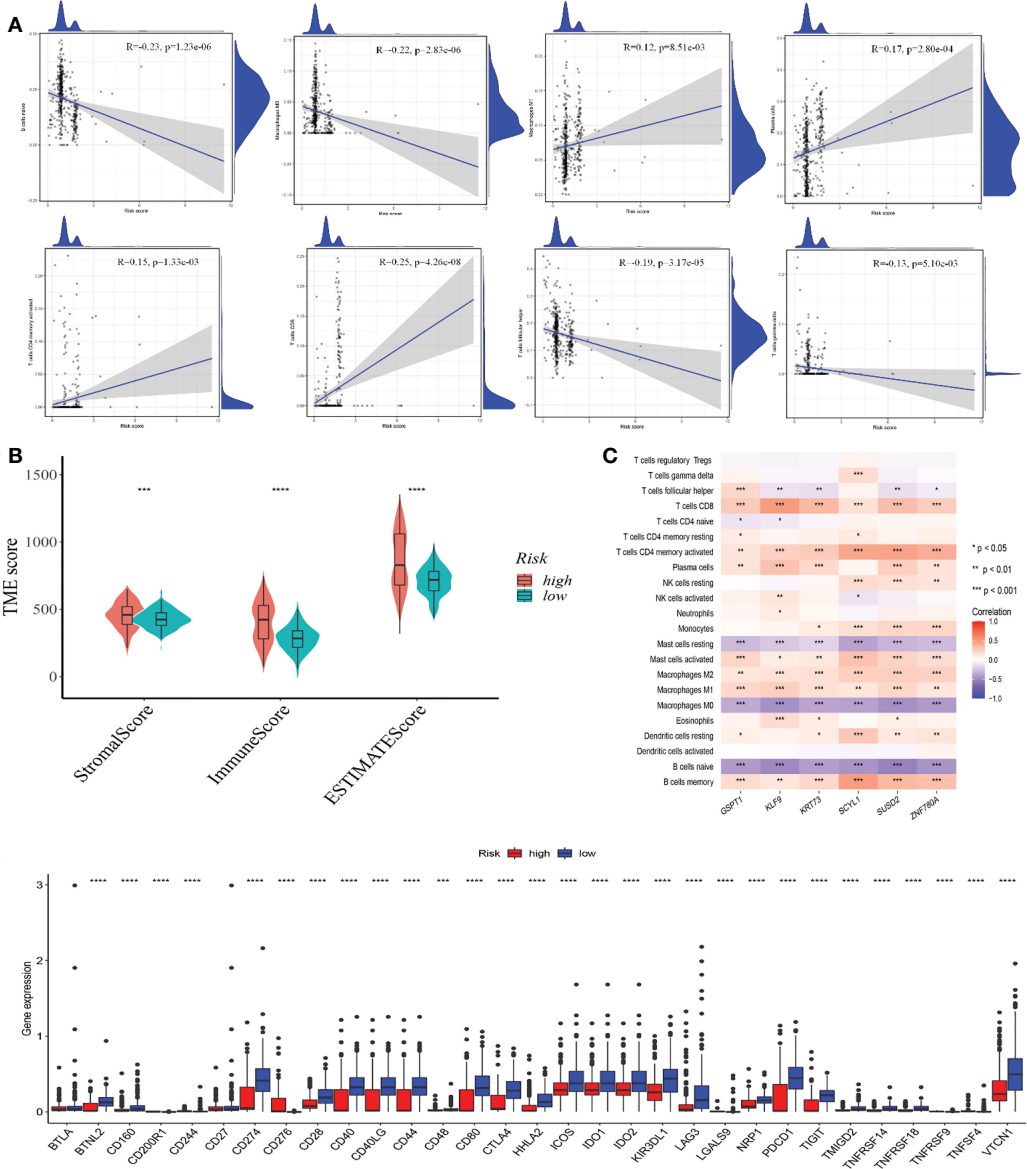
**(A)** TME evaluation and checkpoints between the two classes Correlations between CRG scores and immune cell types. **(B)** Relationships between CRG and immune and stromal scores. **(C)** Correlations between immune cell abundance and seven genes in the proposed model. **(D)** Immune checkpoint expression in high and low-risk groups. TME, tumor microenvironment. *p-value<0.05, **p-value<0.01, ***p-value<0.001, ****p<0.0001.

We used the CIBERSORT method to examine the relationship between CRG score and immune cell abundance. The CRG score was favorably associated with M1 macrophages, plasma cells, activated memory CD4 + T cells, and CD8 + T cells, and negatively associated with nave B cells, M0 macrophages, follicular helper T cells, and gamma delta T cells (**Figure 7A**).

A high CRG score was linked to a high stromal score, immunological score, and estimate score (**Figure 7B**). We also looked at the link between the six genes in the suggested model and the number of immune cells. Most immune cells were shown to be highly associated with the six genes (**Figure 7C**). In addition, we looked at the relationships between immunological checkpoints and our risk model. **Figure 7D** indicates that 32 immunological checkpoints, including PD-L1 and CTLA-4, were expressed differently in the two groups.

### 3.8 Relationship between CRG score and MSI

**Figure 8:**
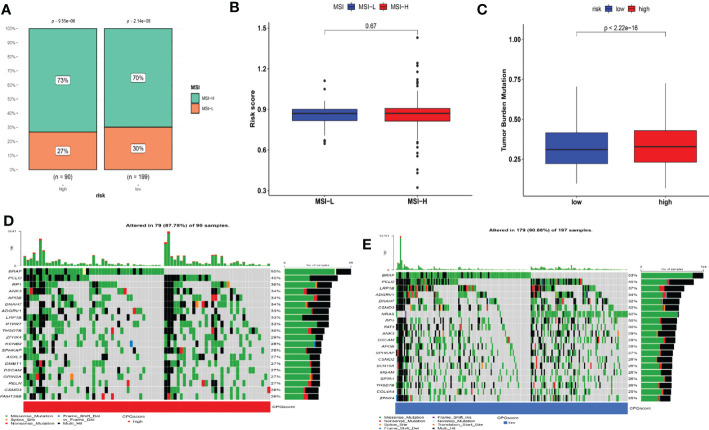
Detailed CRG score analysis in melanoma. **(A–B)** Relationships between CRG score and MSI. **(C)** TMB in various CRG score groups. **(D–E)** A waterfall plot of somatic mutation features with high and low CRG scores. Each column represented a different patient. The upper bar plot depicted TMB, and the number on the right indicated the frequency of mutation in each gene. The proportion of each variant type was shown in the right bar plot. MSI, microsatellite instability; TMB, tumor mutation burden.

Correlation studies indicated that a low CRG score was strongly connected to MSI-L status, while a high CRG score was related to MSI-H status (**Figures 8A, B**). We conducted survival analysis in the MSI-L and MSI-H groups to assess the impact of MSI status on OS in patients with melanoma. Although not statistically significant, the MSI group had a propensity for longer survival (p = 0.33; **Figure S9**).

### 3.9 Drug susceptibility and mutation analysis

**Figure 9:**
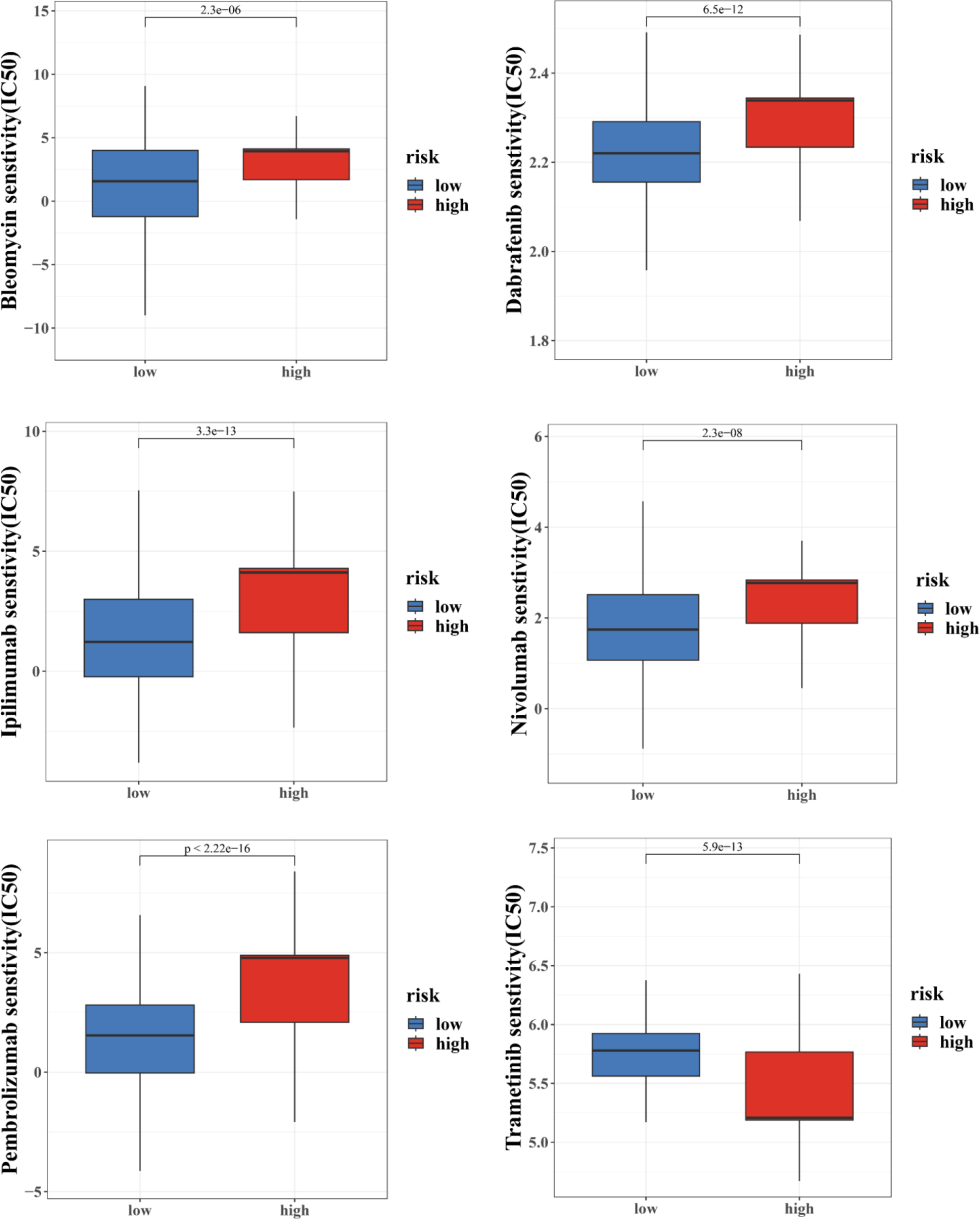
Medication sensitivity analysis based on the risk score.

Moreover, we also computed the tumor mutation burden (TMB) score for each SKCM subject in the two risk groups. Our study of the mutation data from the TCGA-SKCM cohort revealed a high TMB in the high-score group than in the low-score group (**Figure 8C**. The BRAF, PCLO, RP1, ANK3, APOB, DNAH7, ADGRV1, LRP1B, PTPRT, and THSD7B were the top 10 mutant genes in the high- and low-risk groups (**Figures 8D, E**). Patients with a low CRG score exhibited significantly greater rates of BRAF, PCLO, and LRP1B mutations than those with a high CRG score. The mutation levels of APOB and DNAH7, on the other hand, were exactly the reverse (**Figures 8D, E**). We next chose chemotherapeutic medicines, BRAF/MEK inhibitors (Dabrafenib, Trametinib) and immunotherapy drugs (pembrolizumab, nivolumab, ipilimumab) that are already used to treat melanoma to assess the sensitivities of individuals in the low- and high-risk categories to these medications. Interestingly, we found that the patients in the high CRG score group had higher IC50 values for Dabrafenib, pembrolizumab, nivolumab, ipilimumab, Bleomycin (**Figure 9**). However, patients in the high CRG score group had lower IC50 values for Trametinib.

Results for the other chemotherapeutic medicines are given in **Figure S10**. Together, these results showed that CRGs were related to drug sensitivity.

### 3.10 Creating a nomogram to predict survival

**Figure 10:**
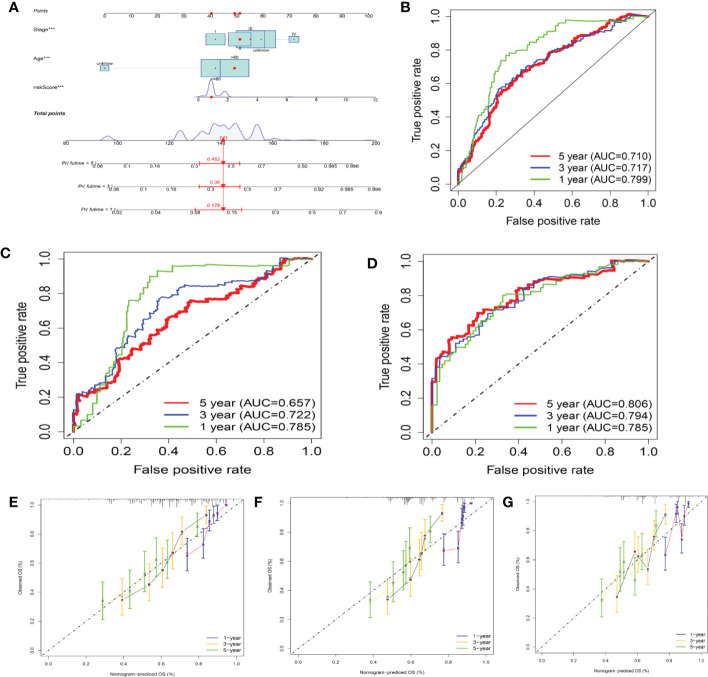
Building and validating a nomogram **(A)** Nomogram for forecasting melanoma patients’ 1-, 3-, and 5-year survival in the training set. **(B–D)** ROC curves in the training, testing, and GSE65904 sets for forecasting the 1-, 3-, and 5-year ROC curves. **(E–G)** Nomogram calibration curves for forecasting 1-, 3-, and 5-year OS in the training, testing, and GSE65904 sets.

In this study, a nomogram integrating the CRG score and clinicopathological characteristics was developed to predict 1-, 3-, and 5-year OS rates in patients with SKCM (**Figure 10**). CRG score, age, and patient stage were among the predictors. Our AUC trials on the nomogram model revealed greater accuracy for OS at 1, 3, and 5 years in the training, testing, and one external validation set (**Figures 10B–D**). Our comparison of the nomogram’s prediction performance with that of the age, gender, tumor location, and stage in the three sets (**Figure S11**) revealed that the nomogram had 1-, 3-, and 5-year AUC values in the training set of 0.799, 0.717, and 0.710, respectively, whilst those of the age, gender, tumor location, and stage were lower (**Figures S11A-C**). The 1-, 3-, and 5-year AUC values of the nomogram in the testing set were 0.786, 0.722, and 0.657, respectively, whereas those of age, gender, tumor site, and stage were reduced (**Figures S11D-F**). Interestingly, the AUC values of the nomogram in two external validation sets (GSE65904) were greater than those of the age, gender, tumor site, and stage (**Figures S11 G-I**). The calibration graphs that followed showed that the suggested nomogram performed similarly in both the training and testing sets compared to an ideal model (**Figures 10E–G**).

### 3.11 The pan-cancer analysis of the proposed model

**Figure 11:**
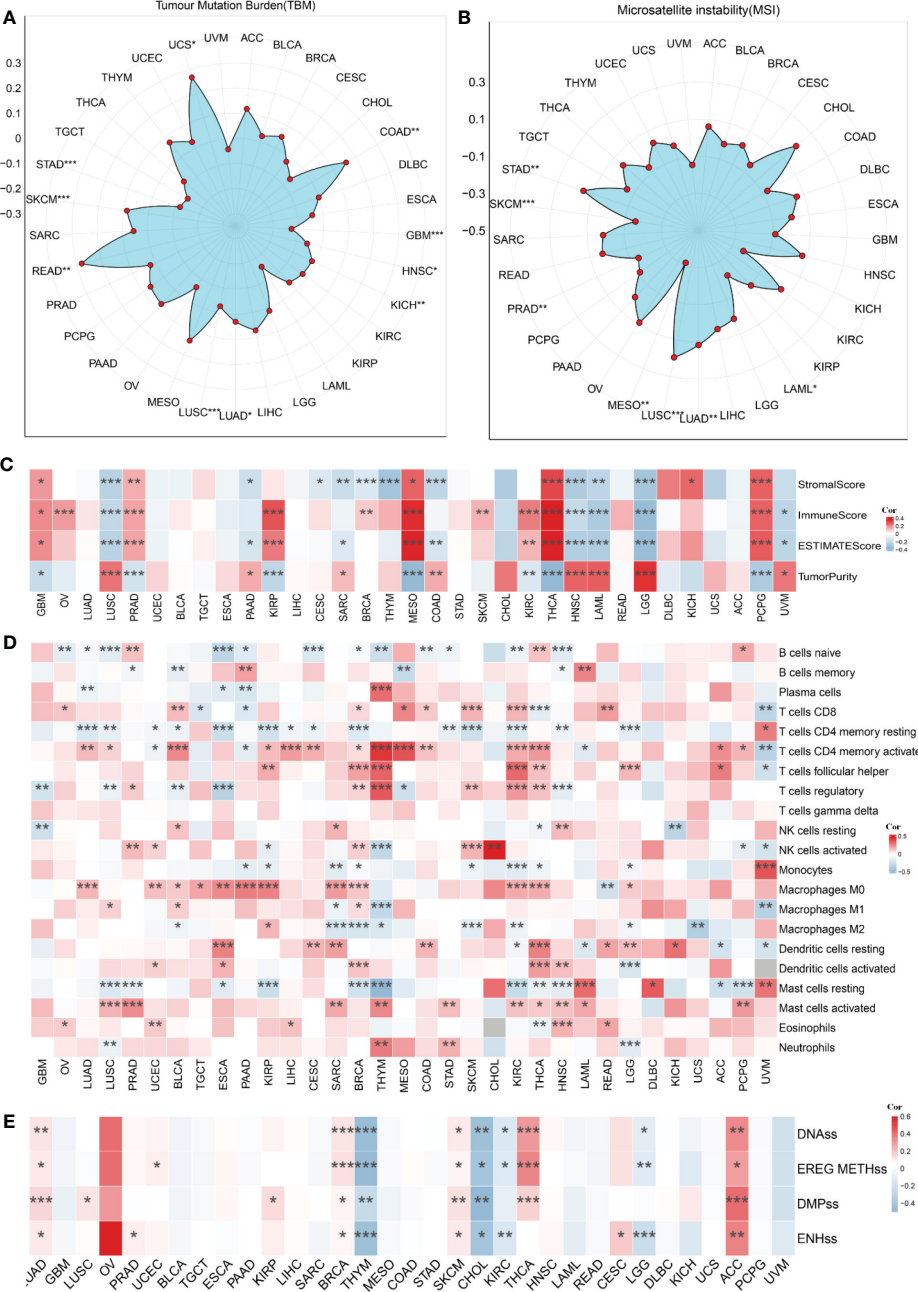
The pan-cancer analysis of the risk score model. **(A)** Tumor mutation burden (TMB), **(B)** microsatellite instability (MSI), **(C)** Tumor purity, ESTIMATES score, immune score, and the stromal score of 33 types of tumors. **(D)** The TME-infiltrating cell of 33 types of tumors. **(E)** The stemness index difference of 33 types of tumors. *p-value< 0.05, **p-value< 0.01, ***p-value< 0.001.

We used pan-cancer analysis to assess the similarities and distinctions of the risk score model across malignancies (**Figures 11A–E**). TMB and MSI were carefully examined across malignancies. TMB was connected with the risk score in COAD, GBM, HNSC, KICH, LUAD, LUSC, READ, SKCM, STAD, and UCS (P<0.05, **Figures 11A**). MSI has a presence in LAML, LUAD, MESO, PRAD, SKCM, and STAD (P<0.05, **Figures 11B**). In addition, we computed the relationship between the risk score and 22 indices of immune cell infiltration and stemness. The outcomes are shown in **Figures 11C–E**.

### 3.12 Validation of RNA expression in A375 and HaCaT cells

**Figure 12:**
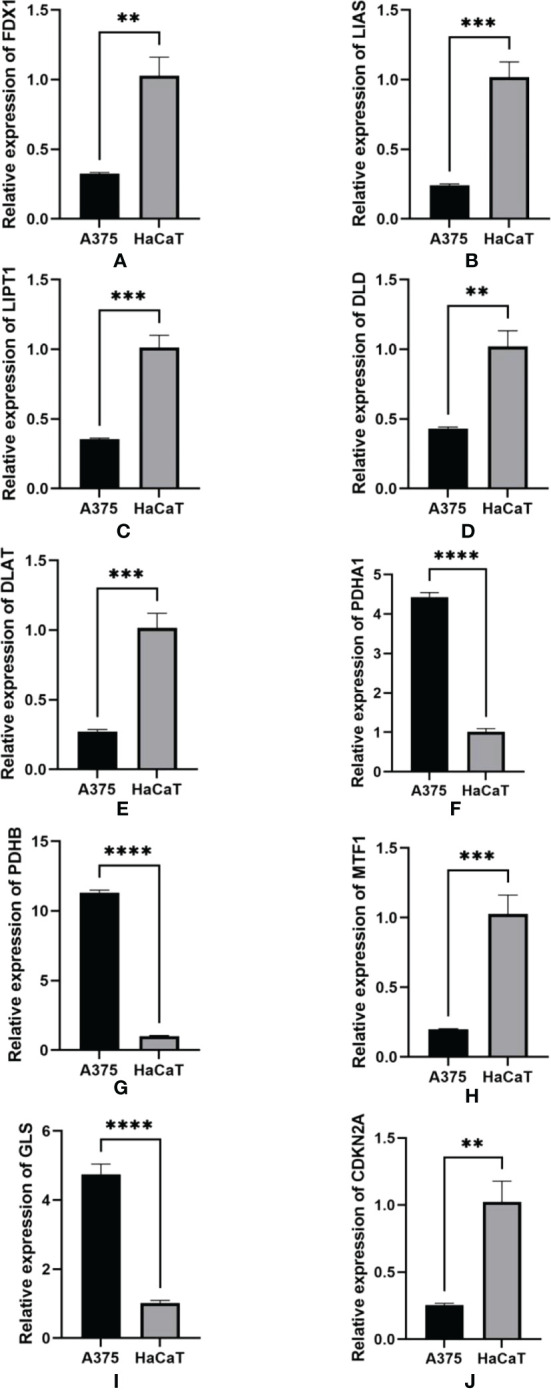
qRT-PCR was detected for the expression of RNA in A375 cell lines and HaCaT cell lines. **(A)** FDX1. **(B)** LIAS. **(C)** LIPT1. D.DLD. **(E)** DLAT. **(F)** PDHA1. **(G)** PDHB. **(H)** MTF1. I.GLS. **(J)** CDKN2A. GAPDH was used as an internal control. **p-value<0.01, ***p-value<0.001, ****p<0.0001.

RT-qPCR was used to detect the RNA level in human melanoma cells A375 and human normal cells HaCaT for validation work of the expression of RNAs identified in the risk model. A375 is a highly differentiated human malignant melanoma cell line and HaCaT is human normal skin immortalized keratinocyte-forming cell line from non-tumor tissues. The results of RT-qPCR in **Figure 12** were consistent with biological analysis. We found that the expression of FDX1, LIAS, LIPT1, DLD, DLAT, MTF1, and CDKN2A were significantly lower in melanoma than in normal tissues. However, the expression of PDHA1, PDHB, and GLS was significantly higher in melanoma than in normal tissues.

## 4 Discussion

Melanoma development is multifactorial and results from a combination of intrinsic and extrinsic factors. Ultraviolet radiation is considered the most significant contributor to melanoma (15). The clinical presentation of melanomas is diverse, which poses enormous difficulties for early diagnosis (16). Various melanoma detection techniques and treatment approaches are emerging to facilitate the management of melanoma. Dermatoscopy is crucial in the early evaluation and offers a more precise diagnosis for melanoma (17). For metastatic melanoma, complete surgical resection can improve the prognosis of melanoma patients to some extent. Studies showed that melanoma patients have significantly improved long-term survival after receiving integration of systemic medical therapy and surgical resection (18). The immune-based interventions have been proven to increase patients’ survival, especially for patients with advanced melanoma. Current immune therapy in melanoma includes vaccines, chemotherapy, and the transfer of adoptive T cells and dendritic cells (19). With improvements in technology, targeted and immune therapies have been continuously evolving. However, there is still a lack of effective molecular targets and accurate classification of melanoma. Therefore, to improve the outcome and prognosis of melanoma more efficiently, more accurate and specific targets and molecular typing need to be explored in greater detail.

Cell death caused by copper was distinct from other cell death, which exerts its effect through targeting lipoylated tricarboxylic acid (TCA) cycle proteins. Copper can directly bind to lipid-acylated elements of the TCA cycle leading to increasing proteotoxic stress, which triggers cell death independent of the apoptotic pathway (20). Mitochondrial signals play an important role in regulating cellular copper content, which can be regarded as the central hub for copper metabolism (21). Mitochondrial glutathione is a natural intracellular copper chaperone, which may decelerate Cuproptosis *via* suppressing lipoylation and promoting DLAT (one of the TCA cycle components) oligomerization (22). In the animal model of Wilson disease, a high level of copper accumulated in mitochondria can disrupt the integrity of mitochondrial membranes, deplete glutathione stores, and increase oxidative stress. Copper toxicity is also associated with the disruption of iron-sulfur (FeS) containing enzymes. The damage to the FeS of mitochondrial ferredoxin can cause growth-inhibitory effects in the downstream signaling pathways. In addition, FeS-targeted damage caused by Copper overload outside mitochondria is also an important part of Copper toxicity in cells (23). There exist some studies investigating the relationship between Cuproptosis and hepatocellular carcinoma, clear cell renal cell carcinoma, lung adenocarcinoma, and bladder cancer (24–27). However, the specific relationship between Cuproptosis and melanoma remains elusive. Therefore, in the present study, we constructed a comprehensive analysis of CRGs in melanoma. Separately, CRG scores were developed to predict the Cuproptosis subtype, prognosis, and outcome of treatment in melanoma.

In the present study, we collected the gene expression profiling data of 781 melanoma patients from four GEO melanoma cohorts and TCGA cohorts. We observed a difference in the frequencies of CRGs mutations, indicating tumor heterogeneity among the individual. Based on the previous study (10CRGs), we conducted univariate Cox and KM survival analysis to assess the prognostic value of the 10 CRGs and utilized a consensus clustering approach to investigate the expression characters of CRGs in melanoma. We find that patients can be grouped into two subtypes that present different prognosis and clinical outcomes. Cluster A has a longer survival time and a lower stage. Because GSVA can be used to investigate the biological molecular mechanism of tumor development and progression, we conducted GSVA analysis to further clarify the potential mechanisms and found that immune-related pathways were significantly enriched in subtype A. Interestingly, we found that the 2 subtypes differed significantly in terms of immune checkpoint expression, immune cell and stromal infiltration patterns, which could reflect the tumor immune microenvironment. Next, we distinguished Cuprotosis pattern-associated DEGs and identified genes related to prognosis using univariate Cox regression in melanoma. To go further, we divided the patients into three gene clusters on the basis of the expression of prognostic CRGs with an unsupervised clustering approach. The analysis showed that gene subtype B was really associated with clinical stage and poor prognosis compared with other gene subtypes.

Next, we constructed a predictive CRG score in melanoma based on the expression of CRGs using the LASSO Cox regression technique and multivariate Cox analysis and split patients into two risk groups according to a median separation. Furthermore, we conducted a comparison with LASSO and other machine methods (elastic net and ridge regression) to further highlight the importance of the LASSO model, and the LASSO model compares favorably with other models (**Table S11**) that we found was in accordance with the prior studies (28, 29). The risk score can effectively predict the prognosis of patients with melanoma. Survival analysis for low- and high-risk groups revealed that there was worse prognosis in the high-risk group compared with the low-risk group. Importantly, the CRG risk score in the present study not only can be regarded as an independent prognostic index for predicting the prognosis of patients with melanoma but also has been correlated with different clinical and histological characteristics. For example, patients in stage IV had higher CRG scores than those in stage I, which was consistent with the previous results. Similar to the discussion in the preceding, different risk groups differed significantly in terms of immune cell abundance and immunological checkpoint expression. We also looked at the links between the two risk categories and MSI. Patients with high microsatellite instability (MSI-H) seem to be more responsive to immunotherapy and may benefit from immunotherapy medications, according to growing data (30). Our investigation demonstrated that a low CRG score was strongly connected to MSI-L status. Because they have a larger quantity of neoantigens, individuals with a high TMB may benefit from immunotherapy (31). Our study showed that the high-score group had higher TMB than the low score group, signaling that immunotherapy might help the high-risk group. In addition, our study suggested that CRGs risk score was associated with drug sensitivity of several medicine that are used in melanoma treatment. In this regard, these medications have the potentials to be selected based on different CRG risk scores in the treatment of melanoma malignancy in the future. Given the limitations of the CRG score’s clinical value in predicting OS in patients with SKCM, a nomogram integrating the CRG score and clinicopathological characteristics was developed. Our study indicated that the nomogram outperformed the age, gender, tumor location, and stage in terms of survival predicting capacity. Finally, the expression of 10 CRGs was validated by RT-qPCR experiments in A375 and HaCaT cell lines with similar trends based on database analysis.

Notably, our study has certain limitations that need to be stated. Firstly, there is a lack of a large sample for validation work. The present study was statistically analyzed by available retrospective data whose results may differ from clinical research. Secondly, further study is required to investigate the molecular mechanisms of genes in our study *in vitro* and *in vivo*. Finally, the majority of results are based on publicly available databases, such as TCGA and GEO, which need to be verified by further experiments.

## 5 Conclusion

In conclusion, our study showed that Cuproptosis may participate in the occurrence and development of melanoma. A novel predictive model was defined to provide insights into predicting melanoma prognosis and characterizing the melanoma immunological landscape, which correlates with melanoma prognosis in TCGA and GEO data. By comprehensive and systematic evaluation of the risk score model, could expand the understanding of prognostic genes and help to develop personalized interventions.

## Data availability statement

The original contributions presented in the study are included in the article/**Supplementary Material.** Further inquiries can be directed to the corresponding authors.

## Author contributions

Conceptualization: BH; investigation: BH and AH; writing original draft: AH and BH; writing review and editing: AH, BH, ZW, MQ, and JZ; visualization: AH; supervision: ZW, MQ, and JZ; funding acquisition: MQ and JZ. All authors contributed to the article and approved the submitted version.
