# Supplementary figures and images for: A novel Cuprotosis-related signature predicts the prognosis and selects personal treatments for melanoma based on bioinformatics analysis
Source: Front Oncol. 2023 Feb 6;13:1108128. doi: 10.3389/fonc.2023.1108128 (PMC9941880; doi:10.3389/fonc.2023.1108128)

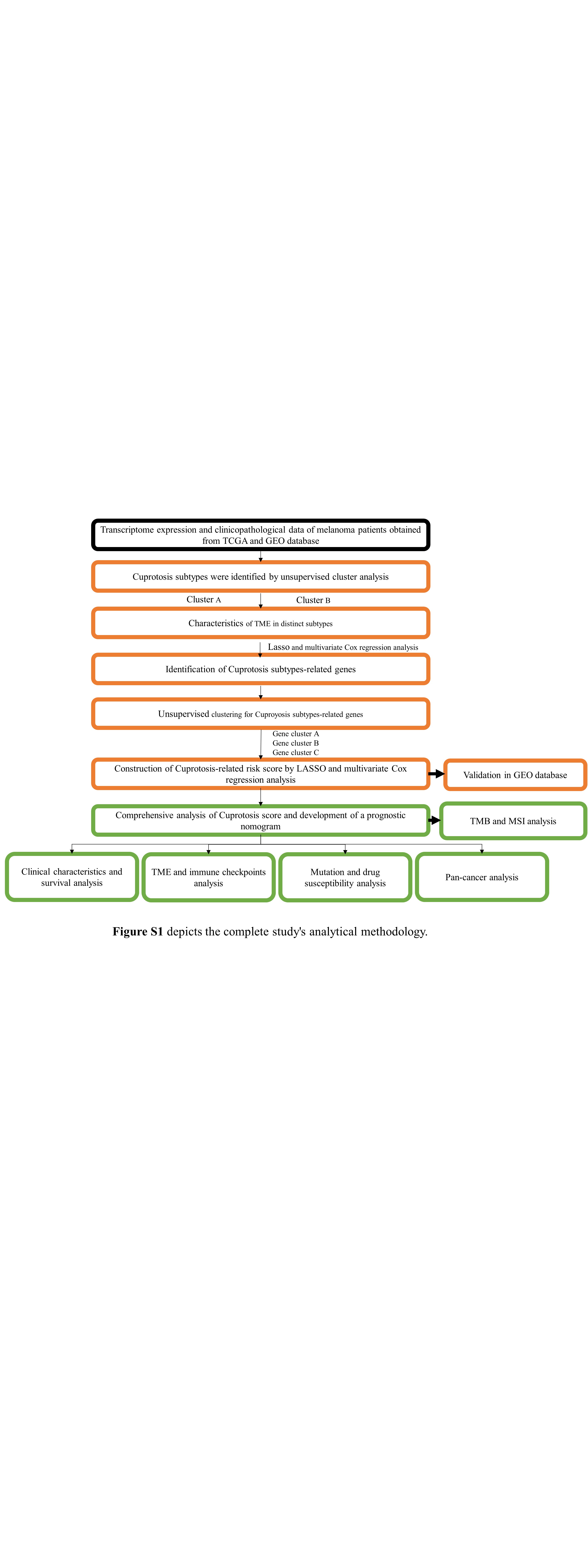

Supplement: Supplementary file 1 [file Image_1.jpeg]

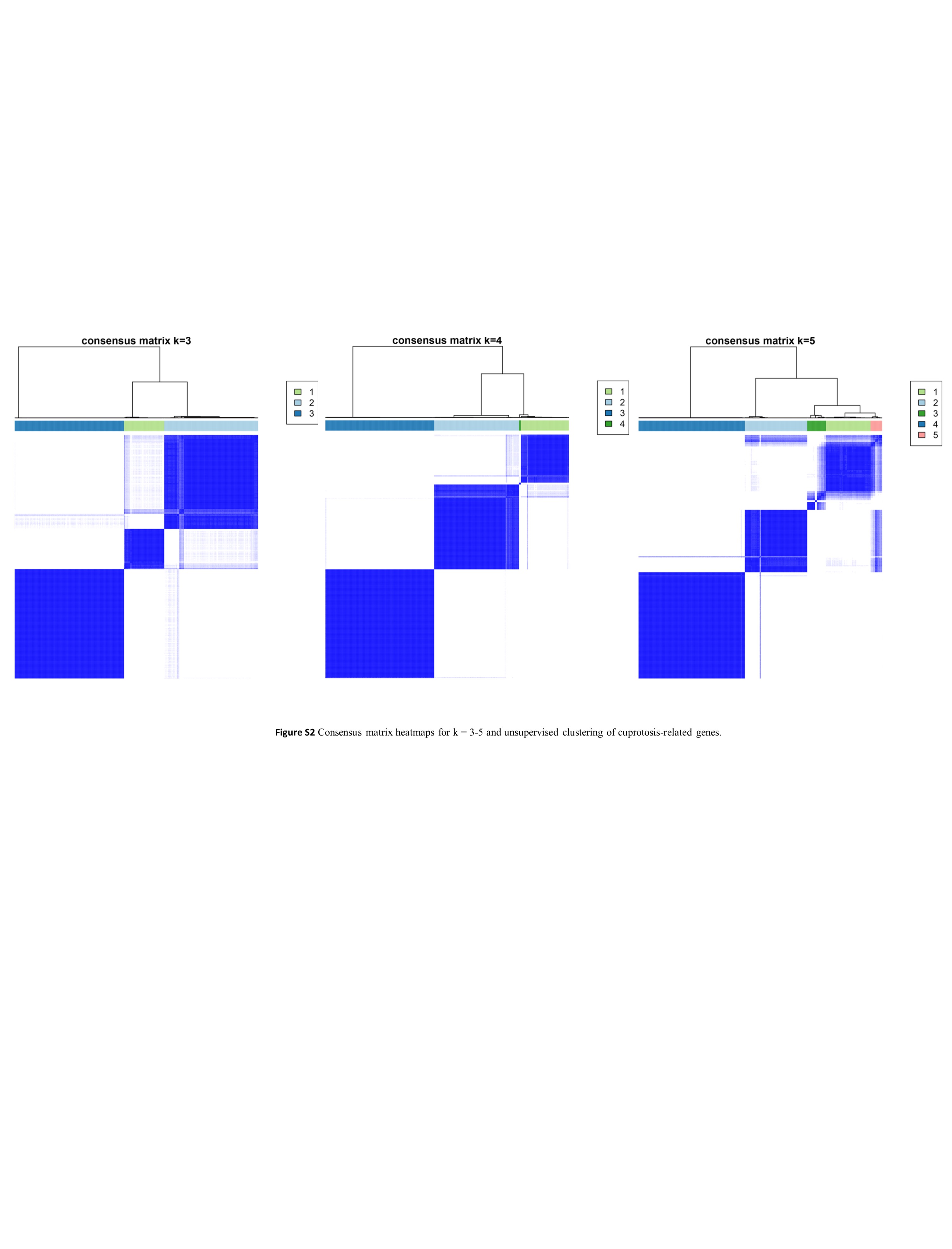

Supplement: Supplementary file 2 [file Image_2.jpg]

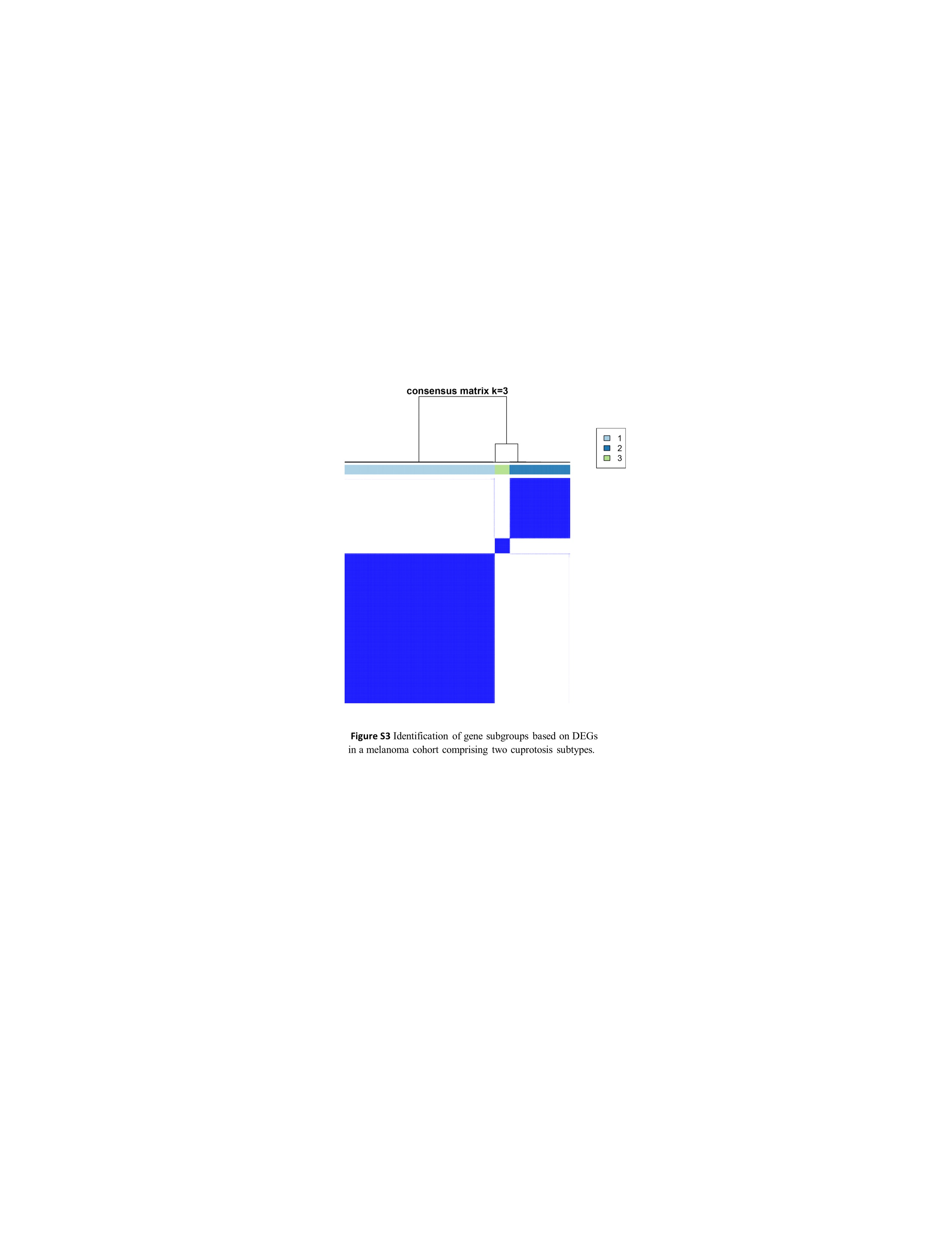

Supplement: Supplementary file 3 [file Image_3.jpg]

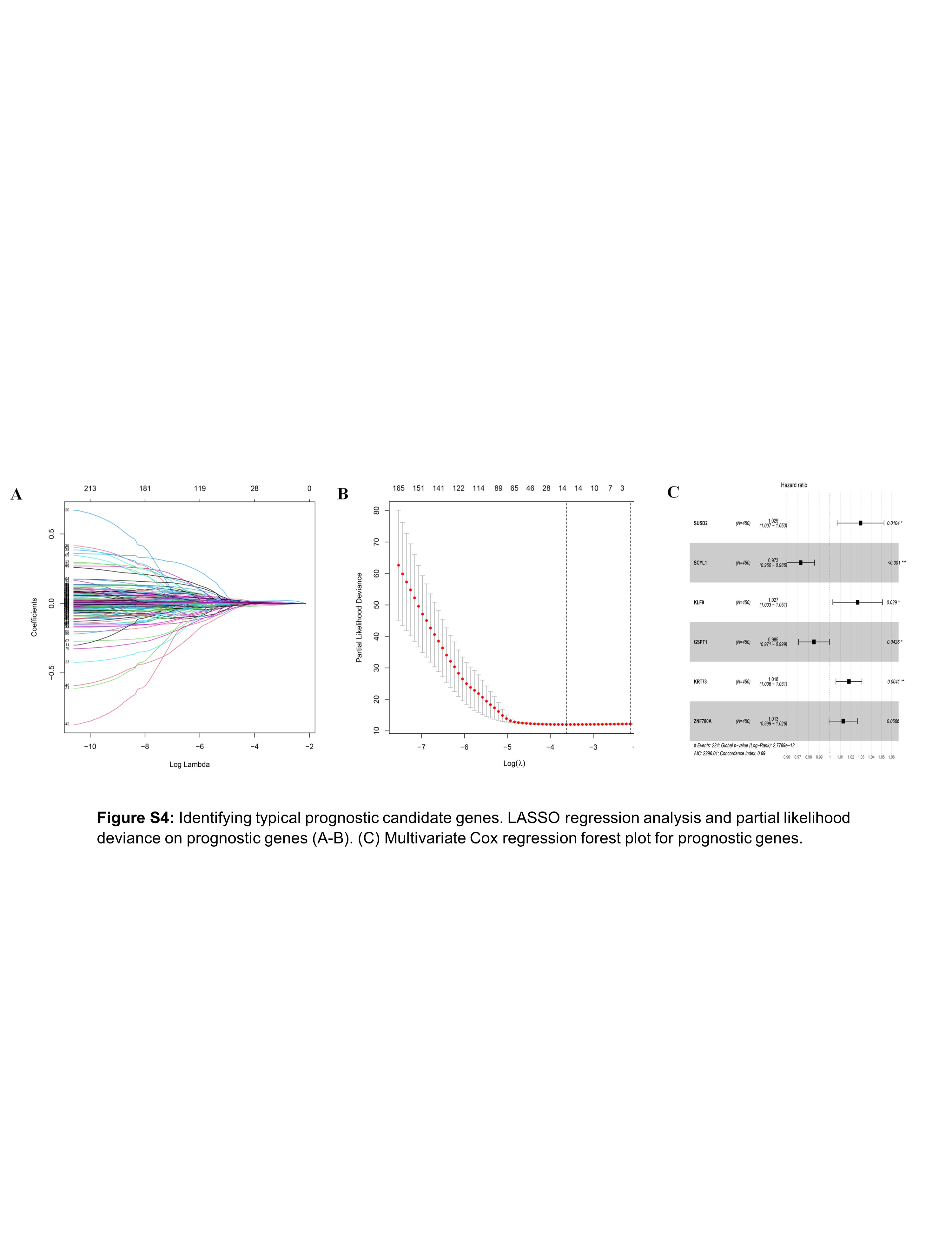

Supplement: Supplementary file 4 [file Image_4.jpeg]

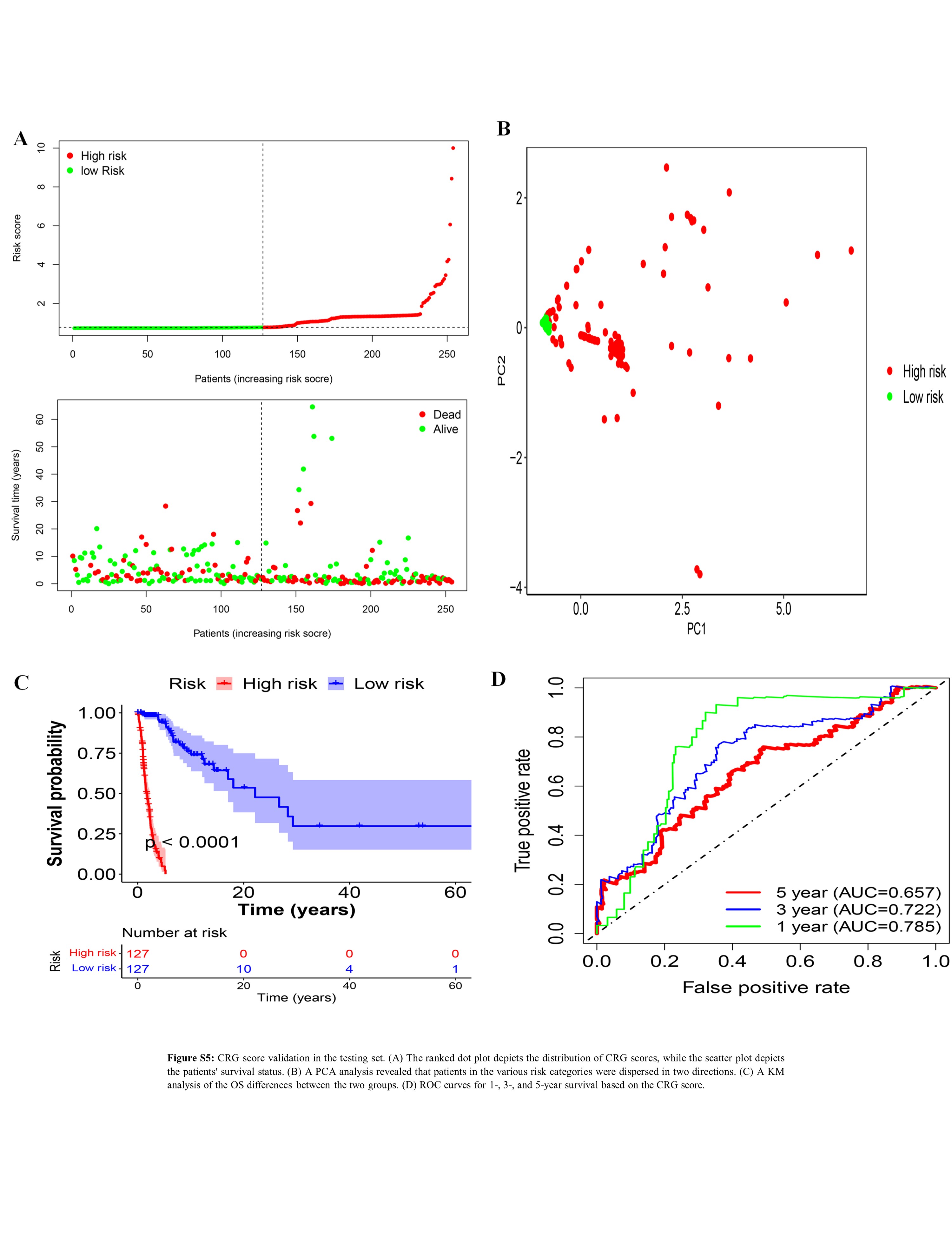

Supplement: Supplementary file 5 [file Image_5.jpeg]

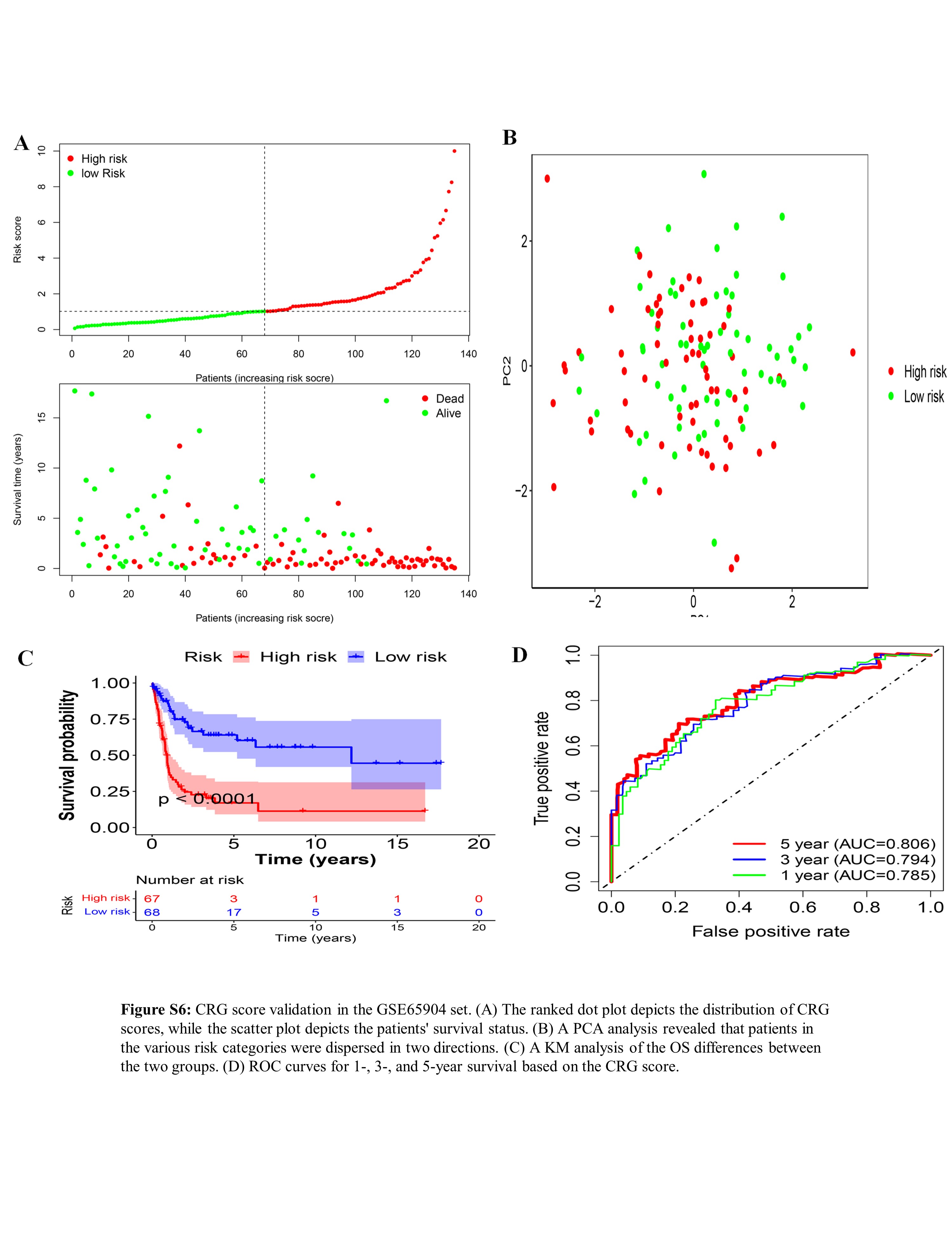

Supplement: Supplementary file 6 [file Image_6.jpeg]

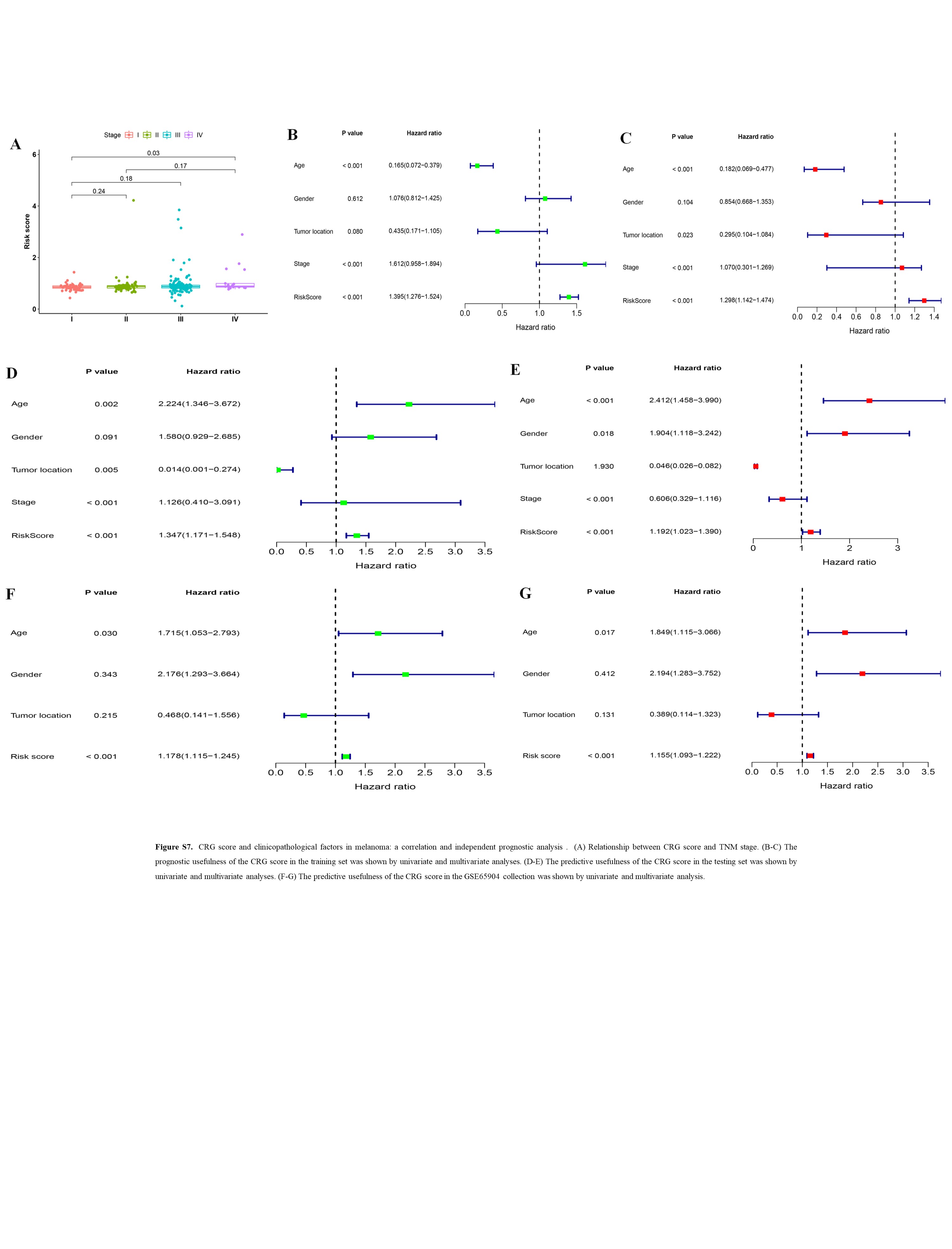

Supplement: Supplementary file 7 [file Image_7.jpeg]

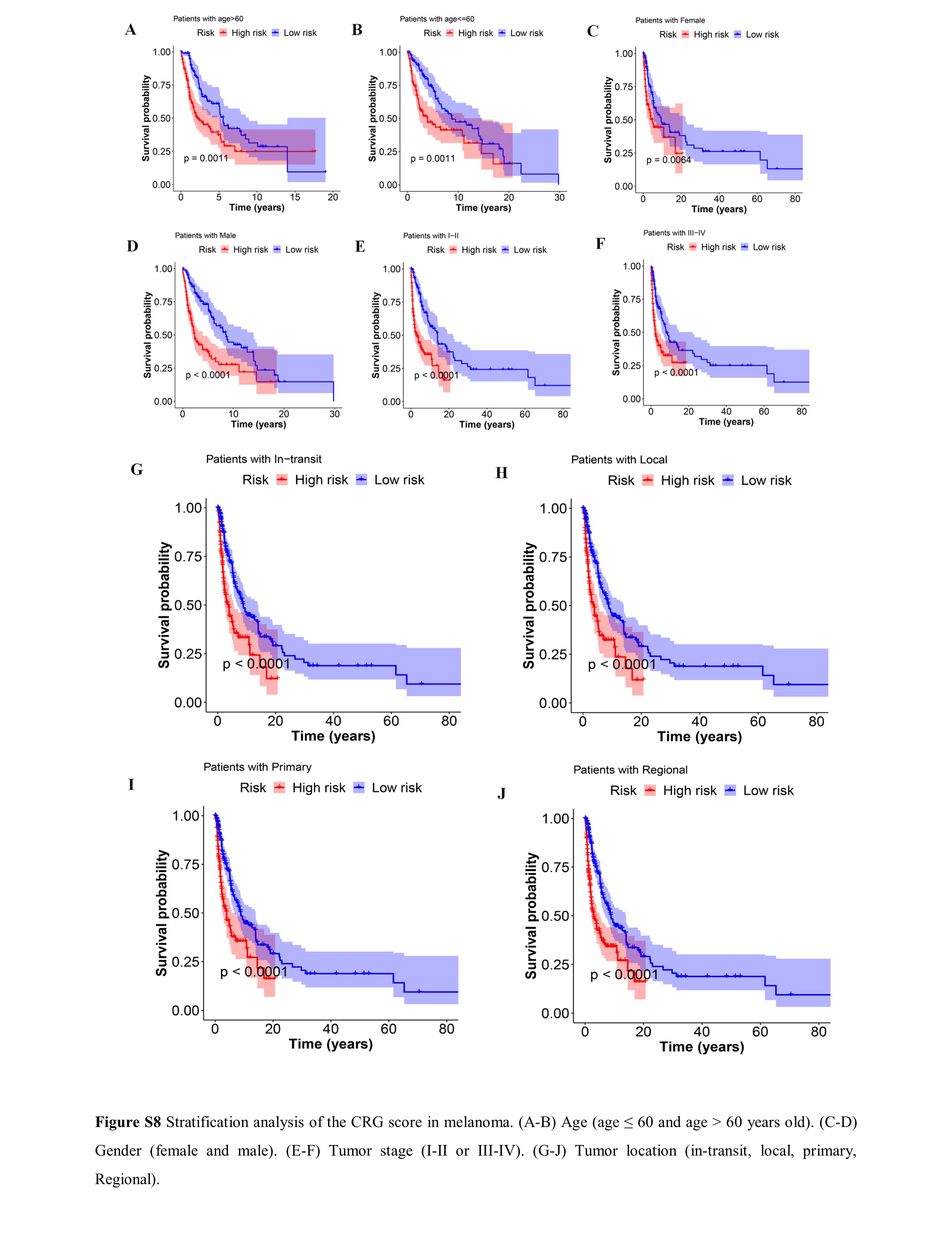

Supplement: Supplementary file 8 [file Image_8.jpeg]

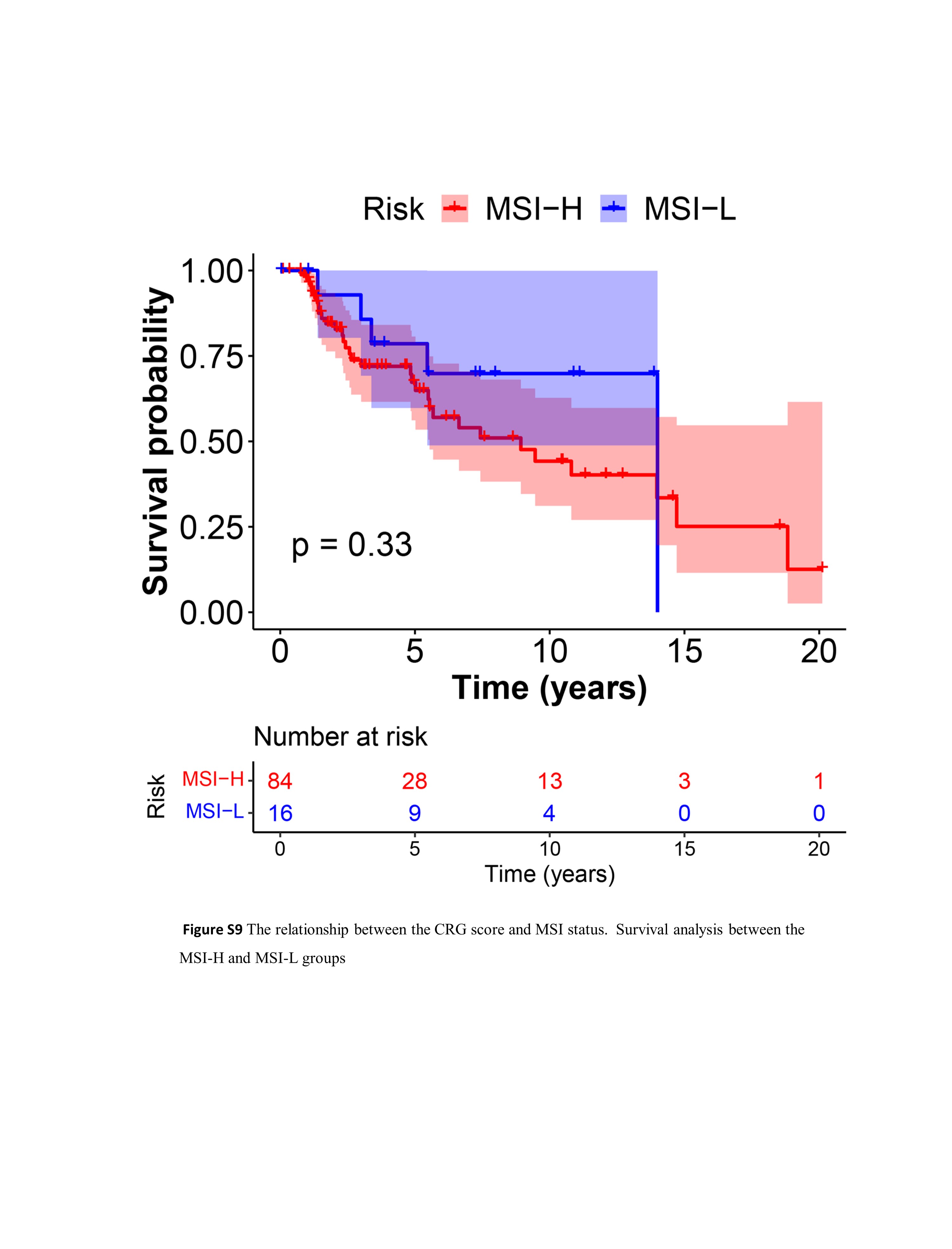

Supplement: Supplementary file 9 [file Image_9.jpeg]

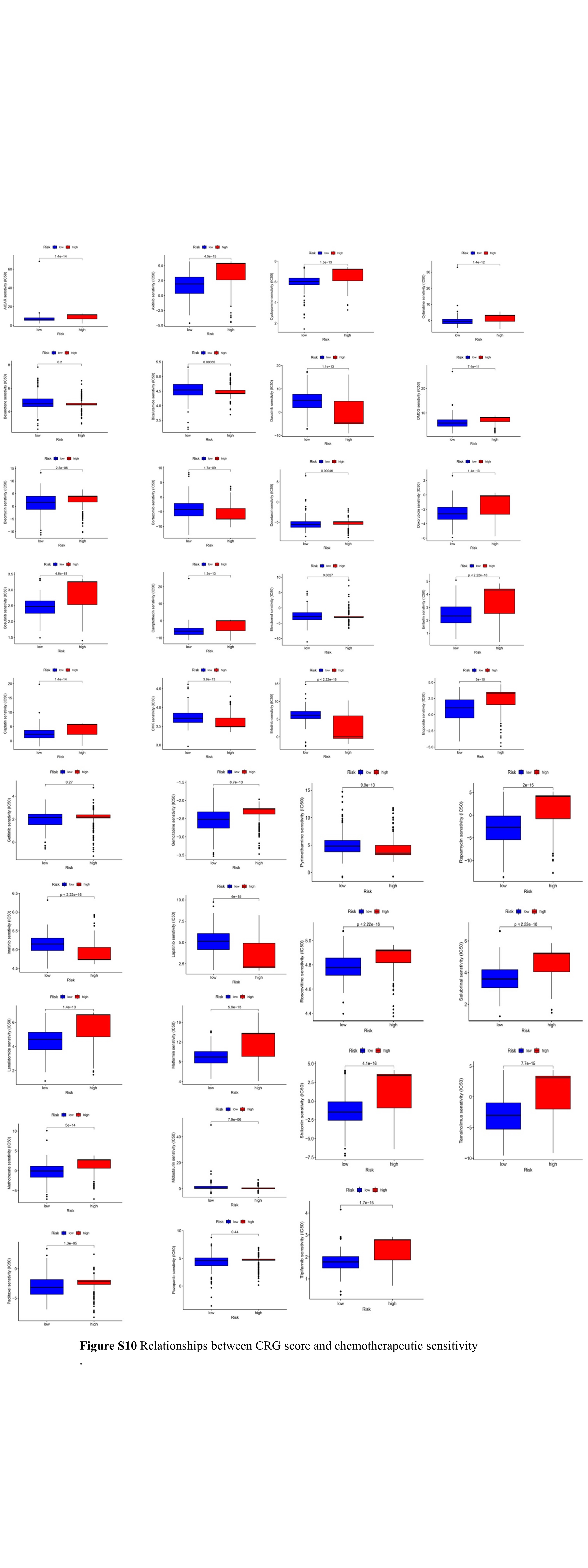

Supplement: Supplementary file 10 [file Image_10.jpeg]

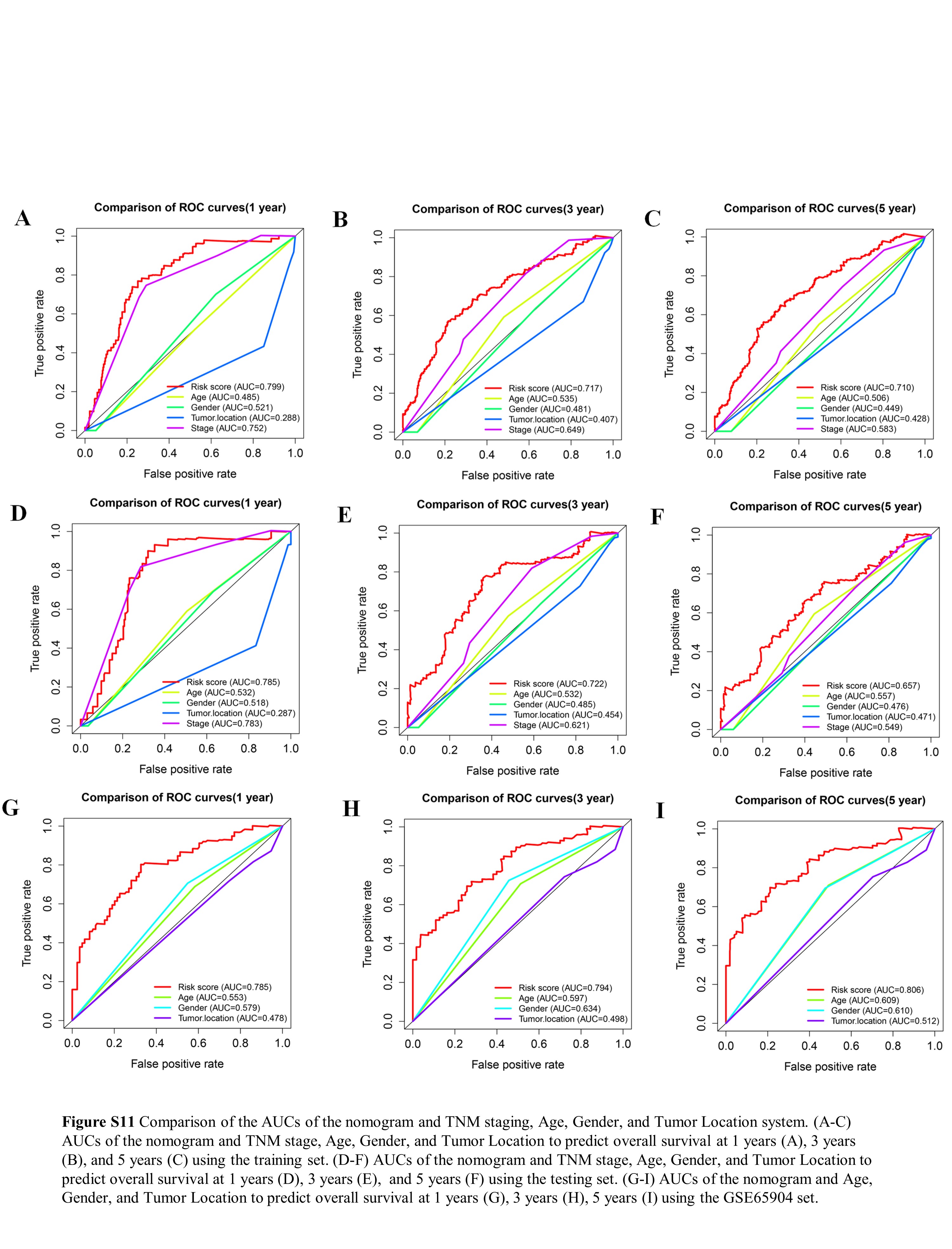

Supplement: Supplementary file 11 [file Image_11.jpeg]
